# Placental Epigenome Impacts Fetal Development: Effects of Maternal Nutrients and Gut Microbiota

**DOI:** 10.3390/nu16121860

**Published:** 2024-06-13

**Authors:** Sanjay Basak, Rahul Mallick, Boga Navya Sree, Asim K. Duttaroy

**Affiliations:** 1 Molecular Biology Division, ICMR-National Institute of Nutrition, Indian Council of Medical Research, Hyderabad 500007, India; sba_bioc@yahoo.com (S.B.); navyasreeboga.nice@gmail.com (B.N.S.); 2 A.I. Virtanen Institute for Molecular Sciences, University of Eastern Finland, 70211 Kuopio, Finland; rahul.mallick@uef.fi; 3 Department of Nutrition, Institute of Medical Sciences, Faculty of Medicine, University of Oslo, 0317 Oslo, Norway

**Keywords:** fetal development, placental epigenome, gut microbiota, immune function, brain development, maternal diet

## Abstract

Evidence is emerging on the role of maternal diet, gut microbiota, and other lifestyle factors in establishing lifelong health and disease, which are determined by transgenerationally inherited epigenetic modifications. Understanding epigenetic mechanisms may help identify novel biomarkers for gestation-related exposure, burden, or disease risk. Such biomarkers are essential for developing tools for the early detection of risk factors and exposure levels. It is necessary to establish an exposure threshold due to nutrient deficiencies or other environmental factors that can result in clinically relevant epigenetic alterations that modulate disease risks in the fetus. This narrative review summarizes the latest updates on the roles of maternal nutrients (n-3 fatty acids, polyphenols, vitamins) and gut microbiota on the placental epigenome and its impacts on fetal brain development. This review unravels the potential roles of the functional epigenome for targeted intervention to ensure optimal fetal brain development and its performance in later life.

## 1. Introduction

Adequate maternal nutrition and optimal environmental factors support a healthy fetus and reduce the risks of adverse outcomes for the offspring in adult life [1]. Several environmental and maternal factors, including the gut microbiota, regulate the developmental stages of the fetal–placental unit via epigenetic, endocrinal, and other pathways [2,3]. Maternal, placental, and fetal coordination and dynamic interaction maintain a healthy pregnancy. The placenta is an indispensable organ that supplies the fetus with oxygen, nutrients, hormones, and growth factors throughout pregnancy [4]. Therefore, optimizing maternal nutrition and limiting exposure to adverse environmental factors can ensure the healthy functioning of the fetoplacental interface [5]. Several fetal factors influence the delicate epigenetic balance in mammals, including maternal–fetal nutrient resources and maternal gut microbiota [6]. Maternal nutritional factors and environmental exposure can alter the placental epigenome, which affects fetal growth and development. Epigenetics refers to the potent interplay between genetic and environmental components regulating gene expression via mechanisms involving alterations to the DNA coding sequence [7], resulting in all cells in an organism showing phenotypic plasticity without change in their genome. Studies aiming at maternal diet manipulation during pregnancy and examining fetal epigenetics provide the most convincing evidence that prenatal nutrition influences fetal methylation and gene expression patterns [8].

In addition to folic acid’s role in countering the development of neural tube defects, its deficiency first pointed to the fetal nutritional programming hypothesis through epigenetic programming. A diet deficient in methyl donor (restricted B12 and folate) during the critical period of the methylation programming phase, which spans from oocytes to embryonic development, resulted in excess adiposity, dysregulated immune response, insulin resistance, and hypertension in male offspring [9]. Genome scanning has confirmed a widespread change that is connected with the effects of nutritional programming [10]. This nutritional epigenetic programming has also been observed with a prenatal n-3 fatty acid-restricted diet and its impact on the offspring’s phenotype [11]. Maternal n-3 fatty acid restriction typically altered placental epigenetic machinery by changing the methylation pattern [12] and affected gene expression of neurogenesis modulators in the brains of young mice [13]. Maternal one-carbon metabolism is critical for maintaining fetal epigenetic stability and shows intergenerational effects. When pregnant mice were fed a diet low in betaine, choline, and methionine, the *Cdkn3* gene, which encodes for kinase-associated phosphatase (Kap) in the fetal brain, was hypomethylated. Hypomethylation of *Cdkn3* correlated with its increased expression, suppressing cell cycling and reducing neural progenitor cell proliferation in the fetal brain [14,15]. Again, maternal choline intake modified the epigenetic signature of the cortisol-regulating genes in the placenta by hypermethylating the glucocorticoid receptor (NR3C1) and corticotrophin-releasing hormone (CRH) genes [16].

The DNA methylation landscape undergoes considerable changes throughout pregnancy to assist placental function, fetal development, and maternal–fetal interactions in mammals. Soon after fertilization, maternal and paternal genomes exhibit demethylation patterns to erase distinct parental methylation patterns and prepare for early embryonic developmental reprogramming. After the formation of the blastocyst and its implantation in the uterine lining, DNA remethylation occurs to build lineage-specific DNA methylation patterns [17,18]. DNA methylation levels change globally by connecting with the development of implantation and subsequent progress in fetal growth, and they also coordinate in a tissue-specific manner. DNA methylation dynamics reveal that the proportion of the methylome remodeled during developmental phases is more substantial than previously thought [19]. A specific DNA methylation signature is expressed in fetal tissue on the 9th gestational day [20].

Dietary factors can affect the fetal epigenome at multiple stages during development. The stage most susceptible to environmental stressors is embryogenesis, due to its higher DNA synthetic rate, where typical DNA methylation patterns are associated with optimal differentiation and growth of the fetus [21]. However, the precise roles of maternal methyl-enriched diets during pregnancy in modulating DNA methylation are unclear. Dietary methyl sources can alter the epigenome entirely. The apparent mechanism is that the diet changes the ratio of *S*-adenosyl-*L*-methionine to *S*-adenosyl-*L*-homocysteine concentrations in tissues and directly alters the activity of DNA methyltransferase [22]. Food bioactives and phytochemicals also alter the epigenetic landscape of phenotypes. For example, sulforaphane, a cruciferous bioactive, downregulates DNA methyltransferase 1 (DNMT1) expression and induces cyclin D2 (CCND2) demethylation [23]. Genistein (a phytoestrogen in soybeans) also affects DNA methylation by inhibiting DNMT1 in vitro [24]. Genistein, comparable to high-soy diets consumed by humans, fed to pregnant Avy methylation indicator mice resulted in shifted coat color in the offspring, significantly correlated with CpG hypermethylation of the Agouti gene [25,26]. Even though the underlying mechanism of epigenetic perturbation remained elusive, the outcome was similar to the methyl donor-depleted state of the model, indicating the early evidence of Avy allele sensitivity due to dietary changes. The gender-specific effects of micronutrients, including folate, vitamins B12, A, C, and zinc, on lowering methylation levels in DMRs of IGF2R in girls and GTL2–2 in boys were also noted [27]. Moreover, increased expression of several placental methyltransferases was observed [16]. Early-life exposure to dietary methyl group supplementation elevated the epigenetic variation over six generations in isogenic mice [28]. Thus, substantial evidence from models and clinical studies reveals that methyl donors and cofactors in the diet can alter the DNA methylation of the fetus.

In addition to epigenetic methylation contributors, early-life metabolic adaptation of the fetus relies on maternal gut microbiota. The gut microbiota can influence the placental epigenome by producing bioactive metabolites such as short-chain fatty acids (SCFAs), trimethylamine (TMA), and trimethylamine N-oxide (TMAO). Such epigenomic modifications could be responsible for fetal programming [29]. Moreover, gut microbial metabolites can also affect pregnancy outcomes via diverse mechanisms [30]. Gut microbiota can modify the host’s immune and metabolic functions, as demonstrated by their interaction with the epigenome, which indicates the potential involvement of the maternal microbiome and its metabolites in fetal programming through epigenetic mechanisms. Maternal BMI, metabolic disorders, diet, ethnicity, geography, and environmental factors can influence maternal–fetal microbiome composition. For example, during maternal obesity, a change in gut microbial diversity is significantly associated with fetal brain development [31]. Gut microbiota also regulates the onset of pre-eclampsia, a placental disease, by producing a different composition of gut metabolites such as SCFAs, TMAs, TMAOs, and others [32]. Maternal gut microbiota also affect offspring metabolism and immune system [33]. The potential correlation between the gut microbiota and the metabolic requirement of the developing brain has been studied [31,34,35]. A broad perspective on the human brain’s early postnatal development and the involvement of the gut in crucial neurodevelopmental processes is also documented [31,35].

The thrifty phenotype hypothesis relies on early-life metabolic adaptation for the fetus’s survival by choosing an appropriate growth trajectory in response to adverse dietary challenges and environmental exposure. In response to nutrient availability or scarcity, maternal metabolic control minimizes in utero energy expenditure and maximizes the storage of reserve calories that the maternal microbiome might regulate [31]. The imprinted genes, such as IGF2, regulate the development of critical metabolic organs and their functions in controlling metabolic axes in postnatal life [36]. The imprinted genes, expressed from one chromosome in a parent-of-origin manner, depend on the epigenetic modulation of expression control, making them sensitive to environmental changes in utero. Moreover, early fetal growth relies on the activation of imprinted genes since the latter is required to successfully develop both embryo and extraembryonic tissues. Therefore, it is possible that maternal nutrition and gut microbiome, as metabolic modulators, closely interact with the fetal epigenome in determining the growth and development of the fetus. However, there needs to be more knowledge about host nutrition and microbiome in placental epigenomic programming that determines fetal development.

The literature was collated from several databases, including PubMed, Scopus, and Google Scholar, using search terms such as fetal brain, placental epigenome, gut microbiota, maternal diets, pregnancy, and essential nutrients. Several clinical and model-based studies were included for review. This narrative review details the latest updates on maternal nutrients, gut microbiota, and their impacts on epigenetic controls of fetal growth, brain development, and functions.

## 2. Heritability of Diet-Induced Epigenetic Changes: Effects on Fetal Development

Diet-induced epigenetic changes are mitotically heritable; whether changes induced in one generation can persist in the next is debatable. The multigenerational effects of epigenetic changes persist as many as three generations after the dietary change and are considered “transgenerationally inherited” [37,38,39]. Diet-induced multigenerational epigenetic changes were first demonstrated in viable yellow agouti (Avy) mice. Subsequently, several studies have confirmed such epigenetic changes transmitted to the next generations in unexposed offspring. For example, a preconception paternal diet (high-fat or low-protein) results in altered energy metabolism in unexposed rodent offspring [40,41]. Such data demonstrate male germline transmission of diet-induced incorporated epigenomic traits via semen composition, altering offspring metabolic programming.

Multigenerational epigenetic inheritance can be transmitted due to maternal exposure to diverse diets (e.g., a high-fat diet (HFD), caloric restriction, and vitamin D deficiency) during pregnancy. Evidence of multigenerational epigenetic inheritance in humans is limited due to challenges in the duration, costs, and participant retention rates required for longitudinal studies. More importantly, beyond these limitations, humans do not live in an isolated and uncontrolled environment and are exposed to various environmental factors throughout their lifespan, making it impossible to assess direct links between diet and epigenetic outcomes.

Different epigenetic mechanisms may partly explain why dietary factors in critical developmental stages might affect the susceptibility to developing metabolic diseases in adulthood. Fetal epigenetic modifications to the maternal dietary intervention are substantiated by many pre-clinical trials (Table 1). MicroRNAs are adaptable, unique regulators of gene expression that play multiple functions during tissue development and disease. However, the roles of microRNAs in fetal programming are limited. Several in vivo studies have assessed the impact of maternal diet on microRNA expression in the offspring and their connection with predictive cardiovascular risks in later life. Such evidence points to nutritional status during pregnancy possibly influencing the offspring’s epigenome, predisposing them to acquiring altered cardiometabolic risk factors, partly through microRNA action. Therefore, therapeutic intervention with microRNAs can unveil novel strategies to combat “later-in-life” effects of adverse nutritional exposure during critical development stages.

Chen et al. studied DNA methylation in mouse placentas exposed to caloric restriction or ad libitum diets [50]. In addition, several differentially methylated genes were identified as imprinted genes, indicating that imprinted loci could be more susceptible to dietary changes. The comparative profile and the sequence of cardiac miRNA expression in baboon fetuses born to (HFD)/high-fructose-fed dams for four months before pregnancy [52] showed a significant reduction in fetal weight and an increased brain and thymus weight, which was associated with 55 upregulated and 25 downregulated (out of total 80) differentially expressed miRNAs. Maternal HFD/high-fructose diet modulates the expression of several miRNAs that are altered in adult cardiac impairments, such as miR-499, miR-21, and miR-143 [53], in heart failure, such as miR-223 and miR-21 [54], and in myocardial infarction, such as miR-451, miR-30c, and miR-139 [55]. The resultant fetal cardiac fibrosis showed differential miRNA expression than controls and implicated the development of heart disease due to the programming of heart development. The potential roles of miRNA-29c were studied in the carotid artery remodeling of offspring aged 3 weeks, 3 months, and 9 months born to malnourished (50% energy) dams [56]. Maternal undernutrition increased the expression of the extracellular matrix, such as elastin (ELN) or matrix metalloproteinase 2 (MMP2) and collagen 3A1 (Col3A1) as well as collagen 4A5 (Col4A5) proteins in the carotid arteries. During these changes, the expression of miR-29c was repressed, targeting many of these genes for carotid artery remodeling.

Interestingly, the expression of these proteins was blunted when a glucocorticoid inhibitor, metyrapone, was administered from day 10 of gestation to term. These changes were associated with an increased expression of miR-29c, suggesting that miR-29c is involved in the remodeling of carotid arteries in offspring due to maternal malnutrition. Despite these studies, models using genetically manipulated differentially expressed miRNAs are obligatory to unravel epigenetics’ role in fetal heart development due to maternal overnutrition.

Among several fetal programming theories, Barker’s hypothesis supports the notion that in utero undernutrition could predispose to several diseases in later life. In contrast, gestational oversupply of nutrients also predisposes to developing disorders [57]. However, similar phenotypes in adults may occur due to the programming of different pathways, such as an obesogenic maternal environment with a HFD and IUGR with a low-protein diet. The differential effects of low-protein diets could be due to the mismatch between the intrauterine and postnatal exposure window [58], while a HFD results in the “malprogramming” of the hypothalamus and beta cells [57]. However, the role of miRNAs in modulating fetal programming between these two theories is unclear. IUGR is likely associated with persistent changes in tissue development structure and functions.

In contrast, an obesogenic maternal state could be associated with metabolic reprogramming of lipid and glucose metabolism and with a risk of fatty liver, metabolic syndrome, and insulin resistance [59]. Despite several theories of the effect of fetal programming on epigenetic regulation, deciphering the modulation of specific miRNAs’ roles in the presence of undernutrition or excess nutrients could eventually lead to a working fetal programming hypothesis. As many genes are differentially modulated in both scenarios in different tissues, miRNAs could reveal the underlying mechanism responsible for the observed effects in a given phenotype (for example, cardiometabolic disease) by regulating many transcripts.

## 3. Placental Interactions with Environments: Impacts on Fetoplacental Development

Obesity in pregnancy has various impacts on the morphology and function of the human placenta. During early pregnancy in obese women, the human placenta responds to increased maternal insulin levels. The cellular signaling system likely plays a role in mediating these effects, influencing metabolism, inflammation, and oxidative stress. These alterations in placental function can independently and synergistically affect pregnancy outcomes, potentially interacting with other risk factors [60,61]. The placenta has intricate vascularization for fetal blood supply, necessitating considerable angiogenesis. Suboptimal angiogenesis causes aberrant placental size and vasculature. Term placentas of obese women show increased lipid content, infiltrating neutrophils, foam-loaded macrophages, and elevated levels of pro-inflammatory mediators [62,63]. Maternal obesity-induced metabolic alterations influence gene expression, early placental growth, and further changes in placental structure and function [64]. Therefore, placental dysfunctions may negatively impact fetal growth and development [5,64].

Genetics, food, and lifestyle factors all contribute to suboptimal placental angiogenesis [65]. Abnormal angiogenesis in the placenta may directly or indirectly influence pregnancies, causing, e.g., pre-eclampsia, preterm birth, gestational diabetes mellitus (GDM), and IUGR [5,66]. n-3 fatty acid deficits impair placental transport of fatty acids in pre-eclampsia- and GDM-associated fetuses [67,68]. Several angiogenic growth factors, such as fibroblast growth factor, vascular endothelial growth factor, placental growth factor, angiopoietin-like 4, and docosahexaenoic acid, assist in optimal placentation [69,70,71]. High-fat diets and maternal obesity modify the metabolome and induce early modifications in the placental transcriptome, reducing placental vascularity [72]. A high-fat diet during pregnancy promotes ectopic lipid accumulation, which leads to lipotoxicity and persistent inflammation in the placenta [73].

Furthermore, a HFD causes the placenta to modify its metabolic response and morphology (thickness) by influencing angiogenesis. In vivo data revealed lower placental labyrinth depth and higher insulin-like growth factor 2 (IGF2) expression and its receptor genes in the fetuses of high-fat diet dams [74]. Deficiency in n-3 polyunsaturated fatty acids (PUFAs) is similar to the impaired placental phenotypes caused by a high-fat diet. Obese placentas accumulate lipids due to changes in fatty acid transporter expression, lipoprotein lipase activity, and mitochondrial oxidative metabolism [4,70]. Genome-wide analyses of the epigenome, transcriptome, and proteome suggest that placental lipid transport and metabolism changes occur due to maternal obesity [75,76]. Such changes in the obese placenta affect optimal placental functioning in the transport and metabolism of lipids across the fetal unit [77,78]. The obese placenta notably exhibits increased levels of triglycerides, free fatty acids, cholesterol, and total lipids. Such a placenta facilitates increased lipid storage but with impaired lipid transport to the developing fetus, especially with LCPUFAs, which are critically required for fetal brain development [79]. Optimal LCPUFA supply is crucial for fetoplacental development, and any alterations observed in obesity can harm fetal brain development and performance [80,81]. During maternal obesity, pro-inflammatory M1 macrophages dominate over less pro-inflammatory M2 macrophages and thereby shift the trigger towards a pro-inflammatory cascade. An obese state promotes low-grade chronic inflammation, which may exacerbate immune functions in pathogenic pregnancies, including in pre-eclampsia, by lowering uterine natural killer (uNK) cell populations [82]. Maternal obesity can also, through epigenetic alterations, dysregulate placental endocrine control of leptin and adiponectin systems [83]. Weakening endocrine controls lowers the protective effects of the placenta’s response to hazardous environmental exposure.

Maternal obesity and GDM both influence fatty acid transport throughout the placenta. Obese women with diabetes showed elevated levels of placental fatty acid binding protein 4 (FABP4) and endothelial lipase [84]. Obese placentas, on the other hand, had lower levels of FABP5 and a decreased uptake of n-6 LCPUFAs [62,75]. Obese women’s placentas expressed low and high levels of the fatty acid translocase CD36/FAT [84]. The placenta, when exposed to high insulin during early pregnancy, alters steroid hormones in the mitochondria and impairs energy metabolism. Maternal lipid transport and metabolism influence fetal obesity through placental function. IUGR and GDM impair the placental transfer of maternal lipids. Inadequate placental fat-soluble vitamins and LCPUFA transfers may cause metabolic dysfunction and poor fetal growth. The interaction between ANGPTL4 and lipoprotein lipase in the placenta causes fetal adiposity in GDM [85].

## 4. Placental Epigenome and Birth Outcomes

**Figure 1:**
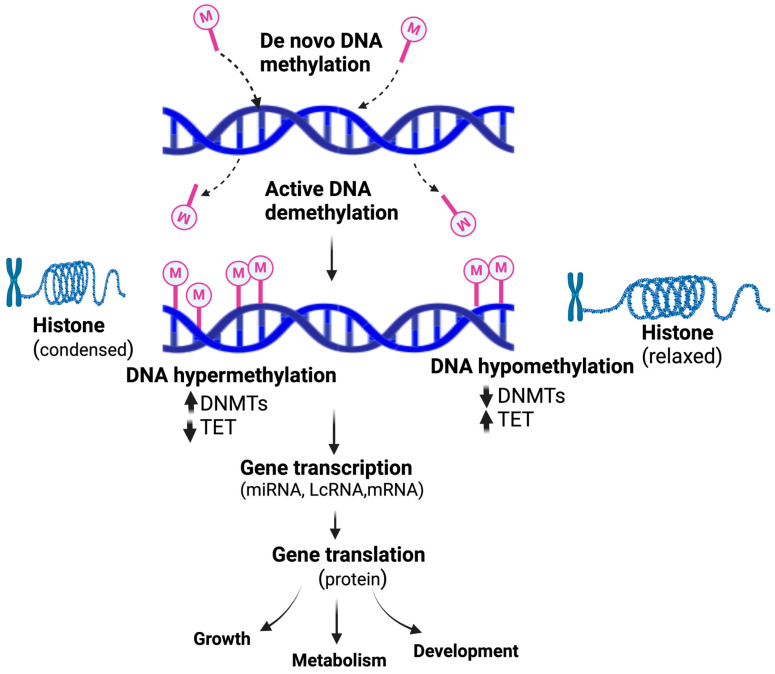
An epigenetic methylation switch modulates gene transcription and the expression of functional proteins involved in the fetus’s growth, metabolism, and development.

Nutrients can impact epigenetics in several ways which modulate gene expression during critical development stages, including histone modifications, DNA methylation, and miRNA expression (Figure 1). Nutrients affect epigenetic changes by changing substrate accessibility, modulating the enzymatic activities of histone deacetylases (HDACs), DNA methyl transferase (DNMT), or histone acetyltransferases (HATs) [86]. The essential intersection of a gene function that could connect metabolism with epigenetics was evidenced by a rise in DNA methylation and a reciprocal reduction in DNA hydroxy methylation in maternal obesity [87].

miRNA expression responds differentially to different nutrients and stimuli in vivo and in vitro. For example, *miR375* and *miR-203* expressions were higher in in vitro culture media enriched with folic acid [88]. The expression of *miR-3079-5p*, *miR-124*, *miR-615-5p*, and *miR-101b* was downregulated, whereas *miR-143* was upregulated in the livers of offspring of choline-enriched dams [89]. miRNAs also regulate the expression of genes that regulate methyl metabolism; for example, *miR-125b* targets DNMT3b in vascular smooth muscle [90], whereas *miR-29b* and *miR-22* control *Mthfr* and *Mat1* gene expression in rats [91]. DNA methylation has epigenetically regulated the expression of several miRNAs, including miRs 375, 149, 27b, 196b, 203, 375, and others [88,92,93,94]. DNA methylation of *miR 1451–5p* inhibits its expression [94], indicating feedback regulation also noted by epigenetic control. Despite limited evidence that integrates exposure and outcome via epigenomic analysis, the advancement of the omics approach has opened up several possibilities to investigate the interplay between exposure, epigenome, and birth outcome. Recent emerging data utilize the placental epigenome to decode biological insight into perturbations related to birth outcomes such as IUGR, preterm status, and others.

**Figure 2:**
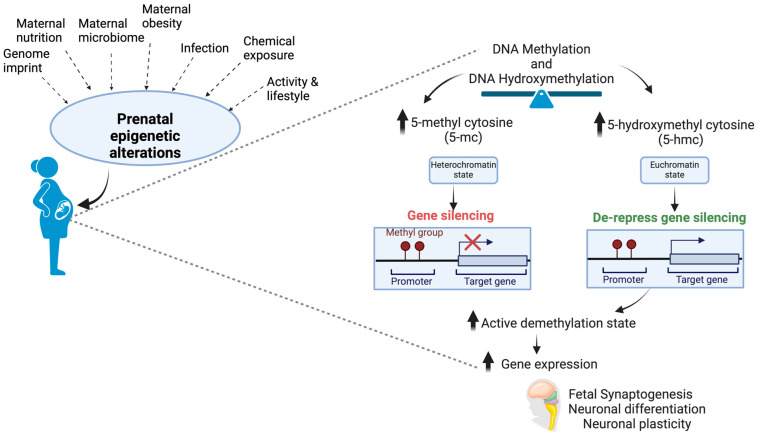
Fetal brain development is also regulated in utero by epigenetic alterations of DNA methylation and hydroxymethylation, which are primed by several factors associated with prenatal exposure.

For example, the association between the placental epigenome and birth outcomes attempted to reveal exposure–outcome pairings concerning birth weight [95]. The methodologies used to examine exposure–outcome pairings include (a) statistical correlation with a subset of target genes, (b) identification of genome enrichment by weighted gene co-expression network analyses (WGCNAs), (c) identification of CpG overlaps with genes associated with exposure or outcome, (d) comethylation status of a chromosome in a single sample, and others. The multi-omics approach, which utilizes mRNA and microRNA expression and DNA methylation state in the placenta, has identified a signature biomarker for placental inflammation and birthweight [96]. Out of thirty-two differential miRNAs, six were related to prenatal cadmium exposure, indicating a promising way to analyze the exposure–outcome axis [97]. Nutrients modulate epigenetics by inhibiting activities of the epigenetic enzymes such as DNMT, histone deacetylases (HDAC), or histone acetyltransferases (HAT) or by changing the accessibility of the substrate required for these enzymatic reactions [98]. Epigenetic alterations via DNA methylation and hydroxymethylation can affect fetal brain development as these are modulated by factors associated with prenatal exposure (Figure 2).

## 5. Maternal Dietary Fats and Placental Epigenome

Maternal nutrients, including dietary fats, can induce the epigenetic modification of the placental genome. The placenta is crucial in facilitating normal fetal development by serving as a primary barrier between the maternal environment and the fetus. It regulates critical functions such as hormone production, gas exchange, and nutrient secretion. During a healthy pregnancy, maternal nutrients can alter regular trimester-specific gut microbial changes, which might affect fetal development [99]. Developing fetuses also mediate immune-suppressive responses to maternal cues during gestation, suggesting a linear cross-talk in the maternal–fetal axis [100].

Maternal fatty acid status, including that of n-6 and n-3 long-chain polyunsaturated fatty acids (LCPUFAs), is a potential predictor of fetus growth and development [1,101,102,103,104]. Membrane phospholipid composition can affect neuronal function by altering enzymes, ion channels, membrane receptors, and fatty acid-derived second messengers. n-3 PUFA deficiency during fetal development affected offspring’s adipose browning in mice [105]; however, no such information is available in humans. A HFD during pregnancy can increase the risk of abnormal brain-related behaviors later in the offspring’s life [31,106]. Fat (adipose tissue) is also the target for hormone signaling and nutrient metabolism through epigenetic modification, which regulates fetal growth and development [107].

The promoter methylation of genes such as *IGF2* and *H19* that regulate fetal endocrine status and growth was used as a biomarker for a maternal DHA intervention study in which pregnant mothers (*n* = 131) were supplemented without or with DHA (400 mg per day) from gestational week 18 to 22 until parturition. The differentially methylated region (DMR) of cord blood mononuclear cells showed one CpG in the IGF2 promoter was higher in DHA-supplemented infants than in control infants without any changes in H19 DMR [108]. The DNA methylation states of genes relevant to regulatory T-helper (Th) cells, such as Th1, Th2, and Th17, did not produce any notable changes in the promoter methylation of the genes DHA-supplemented cord blood. Although DHA supplementation did not alter the growth of the infant, it induced changes in the DNA methylation of pro-inflammatory genes and LINE-1 in infants born to mothers who smoked during pregnancy [109].

## 6. One-Carbon Metabolism-Related Nutrients and Placental Epigenome

Emerging evidence reporting the significance of one-carbon metabolism in fetal programming indicates that maternal low vitamin B12 levels correlate with elevated homocysteine levels, predicting an increased risk of offspring adiposity and greater insulin resistance in humans [110]. One-carbon metabolism modulates global and imprinted gene methylation [27,111]. Specifically, the methylation status of imprinted genes like IGF2/H19 DMR with maternal folate supplementation was researched [112]. A higher intake of maternal choline (930 vs. 480 mg/day) elevated placental corticotropin-releasing hormone (CRH) and glucocorticoid receptor (GR) methylation. It lowered placental CRH transcript abundance, denoting a reduced expression of cortisol-regulating genes in the placenta with extra choline supplementation [16]. Dysregulated one-carbon metabolic enzymes can lead to abnormal DNA methylation patterns and elevated homocysteine (Hcy) in plasma, a toxic derivative associated with vascular lesions. Limiting the intake of B vitamins, folate, and methionine during preconception resulted in a change in DNA methylation and elevated blood pressure and insulin resistance in adult male offspring. Additionally, altered DNA methylation in the renin–angiotensin system, phospholipid homeostasis, and mitochondrion metabolism were observed [113]. Vitamin B12 and folate deficiency during gestation and lactation resulted in lowered birth weight, increased central fat mass, myocardium hypertrophy, and liver steatosis in pups [114].

The essential role of choline in fetal brain development is evidenced by studies showing maternal choline deficiency during pregnancy modifies the fetal brain epigenome. For instance, *Angpt2* and *Vegfc* [115], involved in angiogenic signaling, and *Cdkn3* [14], involved in cell proliferation, were hypomethylated in the fetal brain. Further, H3K27Me3 (repressed) and H3K9me2 levels were stimulated in the fetal brain and liver during maternal choline supplementation, while H3K4me2 (active) levels were maximum in choline-deficient rats. Likewise, choline deficiency also alters the methylation and gene expression of imprinted genes like *IGF2* by affecting Dnmt1 activity in the liver [116,117]. Folic acid is an extensively studied micronutrient which is universally adopted for neural tube prevention among the nutrients in one-carbon metabolism. Folic acid is a one-carbon donor for DNA methylation reactions. Folic acid is required to produce S-adenosyl methionine (SAM), a methyl donor in DNA modifications mediated by DNA methyltransferases. SAM is made in the cytoplasm and drives epigenetic regulations [118]. Several studies have reported DNA hypomethylation with folic acid supplementation. In vivo, DNA methylation responses may be delayed due to folic acid intake, and this can be partially attributed to the prolonged replenishment of folate stores all over the body. Pauwels et al. unveiled a potential delay in DNA methylation responses in women under folic acid supplementation before and during gestation [119]. Therefore, nutrients regulating maternal–fetal one-carbon metabolism may be essential in preventing fetal programming and metabolic diseases.

## 7. Vitamin D Levels and Fetoplacental Epigenome

Maternal metabolism during pregnancy undergoes physiological alterations to ensure the development of a healthy fetus. A close correlation between fetal and maternal vitamin D levels during pregnancy testifies to the rationale of vitamin D supply in this critical time. Maternal delivery of vitamin D could significantly affect the offspring’s growth and development in utero and later in life [120]. An observational association study confirmed an association between low vitamin D and adverse pregnancy-related outcomes for both mother and child [121]. Like others, vitamin D also modulates epigenetic pathways. Low vitamin D levels promote inflammation by changing their mediators’ DNA methylation and histone modifications [122]. The complex vitamin D metabolic network comprises many targets, including the vitamin D receptor and enzymatic regulators that directly or indirectly modulate epigenetic control of the respective genes [123].

Conversely, abnormal vitamin D status may promote epigenetic alterations in various genes, including genes that encode its metabolism [124]. However, our understanding of the interrelationships between vitamin D and epigenetic mechanisms is limited. Initial evidence of an association between maternal vitamin D status and epigenetic alterations in the offspring originated from animal experiments. In a mouse model of vitamin D deficiency, DNA methylation alterations were observed in two generations of offspring, revealing transgenerational effects of vitamin D on the fetal epigenome [125]. In a small human study, Jung et al. reported notable modifications in newborns’ cord blood DNA methylation profiles with high vs. low 25-hydroxyvitamin D (25OHD) levels [126]. Similar results could not be repeated in a recent large genome-wide study where the correlation between maternal 25OHD levels at midterm and cord blood DNA methylation profiles were analyzed [127]. In another study, the impact of midterm maternal 25OHD levels on fetal growth-specific gene DNA methylation levels was investigated, and no association was observed [128]. Thus, further evidence is needed to strengthen the relationship between maternal vitamin D levels and the offspring gene methylations.

Epigenetic modifications regulating gene expression directly affect vitamin D status (synthesis and degradation). DNA methylation mechanisms significantly influence the expression of genes; hypermethylation in the promoter region is responsible for gene silencing and reduced gene expression [129]. Hypomethylation of hepatic *CYP2R1*, the gene that encodes 25-hydroxylase to produce 25OHD, could activate the CYP2R1 enzyme and subsequently increase 25OHD levels [130]. On the other hand, hypermethylation and concomitant lower expression of the *CYP24A1* gene [131], which encodes an essential enzyme of vitamin D catabolism, increases 1,25(OH)_2_D levels. Lower expression of placental CYP24A1 could result in less local degradation of 1,25(OH)2D, which would become the reason for better accessibility of 1,25(OH)2D for the fetus. However, maternal vitamin D levels and the methylation of its metabolic genes in offspring must be further investigated.

## 8. Gut Microbiota and Placental Epigenome

The changes in gestation-specific gut microbiota throughout pregnancy are crucial in maintaining fetal and infant growth and development. Changes in gut microbiota occur throughout life and are particularly pronounced throughout fetal development, infancy, childhood, and puberty, when the microbiota gain sexually dimorphic traits, as well as with aging [132,133,134,135]. Maternal gut microbiota in the early postnatal phase influence nervous system-related activities in infants. Recent breakthroughs in metagenomics have revealed that the placenta contains varied microbiota, which have been examined and documented in healthy pregnancies [136]. An altered placental microbiome (dysbiosis) can cause preterm labor, chorioamnionitis, premature membrane rupture (PROM), IUGR, and even postpartum hemorrhage (PPH) [60]. The use of antibiotics to deplete the maternal microbiota during pregnancy led to a shortage of thalamocortical axons, which hindered thalamic axon growth in the fetus and caused changes in tactile sensitivity in adult offspring. Pre-clinical studies have illustrated that maternal microbiota during pregnancy can impact fetal brain development and postnatal behavior [137,138,139]. Targeted restoration of the maternal gut microbiome prevented derangement in fetal thalamocortical axogenesis [139].

In a recent mother–child dyad study (*n* = 1064), a positive correlation was noted between maternal fecal microbial composition during pregnancy and behavior linked to anxiety in two-year-old children. Furthermore, fecal gut microbiota obtained from pregnant mothers whose children exhibited typical behavior showed an enriched microbial alpha diversity and higher levels of butyrate-producing bacteria [140].

The presence of several bacteria in the placenta and amniotic cavity is associated with chorioamnionitis, miscarriage, preterm birth, premature membrane rupture, and stillbirth [141,142]. However, emerging information suggests that the same bacteria may be present in babies without associated problems. As a result, genetic and/or environmental processes may allow for the progression of unfavorable perinatal outcomes caused by germs during a given gestational stage [136]. Placental and amniotic microbiota alterations have been linked to various pregnancy-related diseases, including bacterial vaginosis [136]. Analyses of placental tissue in pathological pregnancies reveal a prevalence of anaerobic germs over beneficial Lactobacillus.

Preterm birth cases show an increased presence of species such as *Prevotella*, *Bacteroides*, *Peptostreptococcus*, *Gardnerella*, *Mobiluncus*, and *Mycoplasma* [143,144,145]. Women with chorioamnionitis exhibit higher proportions of *Fusobacterium nucleatum*, *Streptococcus agalactiae*, and *Ureaplasma parvum* species [136]. Despite their typically low virulence outside the intrauterine environment, these microorganisms can impact placental vasculature when spread hematogenously, modifying endothelial permeability and allowing the entry of other pathogenic organisms, including *Escherichia coli* [60]. Oral bacteria like *Fusobacterium* or *Capnocytophaga* in preterm placenta correlated with periodontal disease developing toward the end of pregnancy [146]. However, it is crucial to note that changes in flora are not solely associated with oral microorganisms. As pregnancy progresses, hormonal variations lead to shifts in gastrointestinal microbiota. Studies demonstrate significant alterations in fecal flora composition from the first to the third trimester, including increased *Proteobacteria* and Actinobacteria and decreased Lactobacillus [60]. Microorganisms from the gastrointestinal tract and oral cavity, particularly Enterobacteriaceae, have been identified in the placental interface. This is linked to heightened immune tolerance during pregnancy, facilitating the movement of bacteria from the gastrointestinal system into the bloodstream and establishing [136] a path through the bloodstream, connecting various organ systems to the uterine cavity. These changes, influenced by the evolving maternal immune environment, mainly manifest toward the end of pregnancy. The modified flora translocates into the bloodstream, reaching the placental and amniotic cavities and inducing a pro-inflammatory microenvironment.

## 9. Maternal Microbiome and Its Impact on Fetal Growth and Development

The relationship between the mother’s gut microbiota and the fetus is a topic of considerable discussion, primarily revolving around the concept of a “sterile womb”. This idea proposes no direct contact between the fetus and maternal gut bacteria, suggesting that the fetus relies on metabolites derived from the maternal gut microbiota. However, emerging evidence indicates the potential translocation of maternal gut microbiota to the uteroplacental unit [147]. Regardless of microbiota in the uterus, maternal gut microbial metabolites play a vital role in supplying energy, nutrients, and essential vitamins like B complex, folate, choline, and betaine. Environmental variables, such as early life stress, can have long-lasting effects on the brain and behavior via the epigenome, which regulates gene expression [148,149]. The metabolic and immunological alterations observed in pregnant women due to these factors induce changes in maternal gut microbiota that initiate in the second trimester and increase throughout the third trimester [35]. A shift towards low microbial alpha diversity marks these modifications (indicating reduced richness and abundance of taxa) and a heightened beta index (indicating increased variability in composition) [150]. This is linked to increased glycogen- and lactose-producing bacteria, decreased butyrate-producing bacteria, diminished diversity, and augmentation in *Actinobacteria* and *Proteobacteria phyla*.

A healthy gut microbiome in humans is dominated by Actinobacteria, Firmicutes, Proteobacteria, Bacteroidetes, Fusobacteria, and other phyla. The Firmicutes phylum comprises genera such as Bacillus, Clostridium, Lactobacillus, Enterococcus, and Ruminicoccus. Bacteroidetes consist of predominant genera such as Prevotella and Bacteroides. The *Bifidobacterium* genus mainly represents the less abundant Actinobacteria phylum [151]. Faecalibacterium, a butyrate-producing bacterium with anti-inflammatory properties and a member of the Firmicutes phylum, experiences a notable decrease in the third trimester of pregnancy. The third trimester shows elevated beta diversity, which correlates with weight gain, insulin insensitivity, and elevated fecal cytokines, indicating inflammation [146,152,153]. Increased Firmicutes levels are related to the rise in the requirement for energy storage.

In contrast, Actinobacteria and Proteobacteria levels have a protective effect on both the mother and the fetus by reducing pro-inflammatory states [153]. These alterations in maternal gut flora match the fetus’ metabolic demands, contribute to fetal body weight gain, and supply glucose, but they also cause maternal hyperglycemia [154]. During a typical pregnancy, the maternal gut’s operational dynamics and bacterial makeup transition in their diversity due to the inflammatory and immune adaptations essential for maintaining pregnancy. These changes are instigated by modified hormonal levels, particularly the pregnancy-specific hormone human chorionic gonadotropin (hCG).

Consequently, hCG regulates the secretion of estrogen and progesterone, influencing the composition of the maternal gut microbiota. Elevated levels of progesterone extend gastrointestinal transit time, a pivotal factor in shaping the composition and functionality of the gut microbiota [155]. The alterations in the gut microbiota that occur during a normal pregnancy are crucial for maintaining maternal well-being and fostering fetal development. Distinctive changes in the composition of maternal gut microbiota are observed in complicated pregnancies, potentially linked to the increase in progesterone levels [156].

Microbial signatures have been associated with embryonic development, influencing the CNS and immune system while delicately balancing health and disease. Microbial gut dysbiosis, characterized by an imbalance in microbiota homeostasis, has significant implications for overall health. In pregnancy, maternal gut dysbiosis denotes a disturbance in the adaptation of the microbiota to the specific conditions of pregnancy, posing risks to both the mother and the fetus. Various factors, including maternal obesity, dietary patterns, stress, inflammation, infection, antibiotics, and antidepressants, contribute to microbial gut dysbiosis in pregnant women. Maternal obesity during pregnancy is linked to an elevation in *Firmicutes*, resulting in an increased ratio of gut Firmicutes to *Bacteroidetes*. This rise in Firmicutes may enhance calorie absorption, potentially contributing to weight gain and correlating with gut inflammation and increased intestinal permeability. Additionally, obesity during pregnancy can induce alterations in bacterial phyla compared to non-obese pregnancies [157].

Gut metabolites derived from the fermentation of dietary fiber, polyphenols, and other bioactives by gut microbiota may contribute to the nutritional programming of fetal growth and development through epigenetic mechanisms. These maternal metabolites traverse from the gut lumen to the bloodstream, gaining access to the fetus through the placenta and reaching fetal circulation, providing the necessary nutrients for fetal growth and development. Furthermore, these nutrients impact gene expression and contribute to fetal brain development.

## 10. Microbiota and Fetal Immune Development

The placental microbiome is distinct, resembles the mother’s oral microbiota, and can negatively impact pregnancy outcomes [158]. Notably, placental membranes act as a protective barrier in the fetal environment and possess bactericidal properties. This is attributed to cells like extravillous trophoblasts, natural killer cells, leukocytes, and macrophages. Despite bacteria potentially breaching this barrier through bacterial ligands, they became non-viable and fragmented upon passage. Additionally, there is a possibility that certain microorganisms may conceal themselves within placental trophoblasts [159]. During gestation in mice, fetal placental vascularization establishes contact with maternal circulation, enabling the transfer of metabolites, such as SCFAs, from maternal gut microbiota to the fetus. These metabolites contribute to the development of the BBB and innate immunity.

Preterm infants have been found to have a fetal inflammatory response syndrome, which is characterized by hypoxic ischemia and higher fetal pro-inflammatory cytokines, as well as myelination failure [160]. Gut dysbiosis in pre-eclamptic patients had increased plasma levels of LPS and TMAO related to inflammation status [161]. Both rodent and clinical data suggest that maternal microbiota during pregnancy can influence the development of fetal innate and adaptive immunity [162]. Exposing non-obese diabetic mice to vancomycin during pregnancy enhanced offspring susceptibility to type 1 diabetes [163]. In mice, a limited volume of pre-B cells was discovered in bone marrow by gestation day 19, while T lymphocytes were detected at birth [164,165]. The immaturity of the immune system in early development aligns with the function of the underdeveloped intestinal barrier, leading to increased antigen passage across the intestine. In humans, mothers provide natural passive immunization during fetal life by transferring IgG antibodies. Microbial antigens from gut microbiota and maternal antibodies are transferred to the fetus through the placenta, triggering immune activation. Free dietary antigens may also traverse the placental barrier [166]. During the prenatal stage, intestinal digestion is minimal, and amniotic fluid components such as proteinase inhibitors influence the luminal environment and the production of antigenic compounds. It has been proposed that maternal microbial metabolites are essential for immunological activities [167]. SCFAs produced by the maternal microbiota during pregnancy have been found to affect T-cell development, intestinal immunity, dendritic cell (DC) activity, and epithelial integrity [162]. Specific neuropeptides, norepinephrine, and vasoactive intestinal peptides play a role in modulating the functions of dendritic cells and cells in the intestinal wall and secondary lymphoid tissues, such as Peyer’s patches. Bacterial DNA transferred from the mother to the fetus’s gut stimulates mucosal immune development and influences the fetal immune system in preparation for the transfer of maternal–fetal microbiota postnatally [168]. In germ-free mice, the development of fetal thymic CD4+T cells and regulatory T cells (Treg cells) is compromised. Still, supplementation with the bacterial metabolite SCFA-acetate rescues this deficiency. Acetate induces the upregulation of the autoimmune regulator, contributing to Treg cell generation. In humans, low maternal serum acetate is associated with preeclampsia, a pregnancy-associated placental disorder affecting maternal cardiovascular risks and weakened immune systems, leading to reduced Treg cells. Maternal immune states in pre-eclampsia are reflected in the fetal immune system, suggesting a role for metabolites produced by maternal microbiota in regulating fetal immunity [163]. A Danish study observed that antibiotic usage during pregnancy altered maternal microbiota and metabolites that correlated with increased risks of immune-related dysfunctions like immune atopic dermatitis in newborns and elevated risk of developing asthma in children 2–10 years old [169]. Maternal microbiome changes during pregnancy have been linked to type 1 diabetes and inflammatory bowel disease [170].

The enteric nervous system (ENS) undergoes development in the fetal window, originating from vagal and sacral neural crest cells. Maternal gut microbial metabolites, particularly SCFAs, regulate fetal ENS. SCFAs, transmitted from the maternal gut through the placenta to the fetus, serve as an energy source, regulate fetal gut epithelium, and influence the development of the fetal neural system, metabolic system, and immune response [171,172]. Studies in germ-free mice demonstrate a significant reduction in the cross-placental transfer of microbial metabolites compared to specific pathogen-free mice, emphasizing the importance of maternal gut microbiota [173]. Maternal gut dysbiosis negatively impacts fetal intestine permeability and integrity [174]. Recent evidence indicates a direct influence of bacterial exposure on fetal gut colonization [175]. The amniotic fluid and placenta exhibit low diversity and richness, and Proteobacteria are prevalent. The gut microbial similarities among the amniotic fluid, placenta, and infant meconium suggest cross-placental microbial transfer and prenatal seeding of the fetal gut. Meconium microbiota exhibit a closer resemblance to the amniotic fluid than to maternal vaginal and fecal microbiota, suggesting seeding from multiple maternal sites, with amniotic fluid contributing significantly [176]. Microbial contact can initiate healthy immune maturation during fetal life [175]. In both rodents and humans, the development of the digestive system aligns with the maturation of the immune system and the ENS. In mice, the intestinal epithelium undergoes the formation of villi and crypt structures, leading to restricted epithelial proliferation and the cytodifferentiation of villi into functional cell types in the small intestine. During this stage, smooth-muscle layers around the gut tube and ENS develop. The motor neurons and sensory lineage are projected into the gut on embryonic day 14 in mice. The formation of the crypt, brush border, and Paneth cells is observed around 14 postnatal days in mice, which completes the intestinal morphogenesis postnatally [177].

Although anatomy and cellular features develop in utero, the ENS becomes functional postnatally in mice [178]. In humans, the development of the digestive tube, a precursor to the gut, begins in the 3rd week of gestation, with crypt formation occurring around the 12th week and intestinal functions developing by the 24th week of gestation. This development coincides with the ENS, which is not fully mature at birth and continues to develop postnatally [179]. Tight-junction proteins, including claudin, control the intestinal epithelial barrier, with claudin expression beginning as early as the 18th week of gestation. The integrity of this barrier is crucial for regulating transport across the lumen and excluding pathogens.

Consequently, intestinal morphogenesis is completed before birth in humans. Mucosal immunity starts developing in the human fetal intestine by 11–14 weeks of gestation, with dendritic cells populating the developing intestine capable of responding to microbial stimuli and initiating T-cell responses. By the 13th gestational week, memory T cells become abundant in the fetal intestine, indicating early immune priming. Specific immunomodulatory microbes with bacterial-like morphology have been identified in mid-gestation human meconium, accompanied by enriched taxa and T cell patterns. Viable bacteria observed in the fetal intestine at mid-gestation can limit inflammatory potential by interacting with fetal immune cell populations. Fetal T cells demonstrate the ability to form memory in the intestine, suggesting that bacterial antigens contribute to T cell activation. These specific bacteria persist under nutrient-limiting conditions, utilize pregnancy hormones for growth, and can survive within phagocytes [180]. In humans, enhanced maternal–fetal macromolecular transfer occurs across the intestines during early life due to augmented endocytic activities of the nascent intestinal epithelial cells. The transfer of IgG is modest throughout the first and second but increases in third trimester [166]. Multiple research studies have identified bacterial DNA in the placenta and amniotic fluid. Investigations in mice reveal similarities between fetal intestine bacterial DNA, placental bacterial DNA, and maternal oral and vaginal DNA, with additional overlap observed in meconium [181]. Intriguingly, the human fetus starts producing meconium as early as 12 weeks of gestation, hinting at a potential exchange of microbiota between the fetal and maternal environments [182].

Maternal stress in pregnancy induces persistent changes in maternal gut microbiota, impacting the placental transfer of nutrients. Notably, male offspring, not females, exhibit significant alterations in neurodevelopment in the hypothalamic and limbic circuits, affecting stress responsivity [183]. These findings demonstrate that the makeup of maternal gut microbiota during pregnancy is an essential contributor to the metabolic programming of offspring [184]. Initially shielded by the placenta, the fetus gains an additional layer of protection through the maturation of the BBB during pregnancy. In rodents, the BBB starts forming around gestational days 13.5–15.5, characterized by a higher concentration of tight-junction proteins and extracellular matrix components than the adult BBB. Notably, in germ-free mice, the BBB appears leaky around gestational day 16.5, emphasizing the crucial role of maternal microbiota in developing a functional BBB [185]. Some efflux transporters capable of excluding poisons are present in humans as early as eight weeks of gestation, and BBB components appear at twelve weeks. It is established that the blood–brain barrier of the fetus during gestation and at newborn remains immature, or “leaky”, making the developing brain sensitive to toxins or medication entering the fetal circulation from the mother [186,187].

Elevated intestinal permeability during early pregnancy is believed to be linked to higher maternal LPS and cytokines at the endometrial level. This phenomenon is thought to enhance the translocation of bacteria and bacterial metabolites from the intestinal lumen into the maternal circulation [150]. Bacteria can reach the placenta through the bloodstream, potentially facilitated by dendritic cells translocating from the gut epithelium to lymphoid organs. Maternal oral bacteria entering the bloodstream are also suggested as a source of fetal microbiota [188]. These early prenatal microbiota may play a role in priming the immune system for the subsequent colonization of microbiota after birth. In the first year of life, the human intestine quickly becomes populated with microbiota, primarily strict anaerobes. By 2–5 years, the microbiota in infants become individually distinct from the composition and diversity seen in adults [189]. The first diffusion of maternal metabolites across the placenta promotes the development of the fetal nervous system and HPA axis. It is followed by gut bacteria penetrating the placenta and transferring it into the fetal bloodstream. Thus, gut microbiota colonization could occur throughout fetal life [186,190]. Some bacteria may enter the fetal gut, but they are insufficient and not unique enough to activate intestinal epithelial tolerance during the first interaction with microbiota after birth.

## 11. Gut Microbiota and Fetal Brain Development

The developing brain is susceptible to adverse changes in maternal–fetal cues but is protected by barrier transporter systems like the blood–brain barrier [186]. Gut microbiota might play a protective role in fetal brain development by controlling immunity and inflammation [191]. The maternal gut microbiome during pregnancy is critical for the development of the fetal central nervous system. Despite the prevailing belief that the gut–brain axis develops postnatally, based on the concept of a sterile womb, there is a growing recognition of a limited number of distinctive bacteria found in fetuses, suggesting their potential role as transitional species facilitating the establishment of a complete microbiota after birth [162,192].

Maternal factors, such as genetics, diet, health condition, stress, and medication, can determine the microbiota–gut–fetal brain axis [193]. Animal and human studies have established a relationship between maternal gut microbiota and fetal neurodevelopment. Rodent studies have indicated sex-dependent impaired prenatal brain development in germ-free mice [194]. The altered gene expression demonstrated by germ-free mice was associated with neuroplasticity, neurotransmission, metabolism in the hippocampus, and thalamocortical neurodevelopment of sensorimotor behavior and pain perception after birth [139]. The development of the human brain and nervous system begins at six weeks, during embryonic development, and extends throughout pregnancy, continuing into puberty and beyond. Specific neurodevelopmental changes involving axonal growth, synapse formation, and dendritic and axonal arborization occur during gestation, culminating in establishing synaptic connections. Maternal gut microbiota regulate metabolites that reach the fetus through transplacental signaling, influencing fetal brain development [139].

Existing research extensively explores the relationship between gut microbiota and the gut–brain axis (GBA) postnatally. Still, this connection needs to be understood during the prenatal period [195]. While the fully functional bidirectional offspring GBA develops after birth, a rudimentary GBA is established in the fetus under maternal gut microbial control. Due to the absence of a functional GBA in fetal life, maternal gut microbiota influence this rudimentarily developed GBA in humans. Newborns are exposed to significant amounts of maternal vaginal, fecal, and skin microbiota during birth, with colonization influenced by the delivery method (vaginal or C-section). Nursing further contributes to the transfer of maternal microbiota to the infant, with the composition influenced by the mother’s health and gestational age. Gut closure, a developmental stage of intestinal maturation occurring around six months, signifies the establishment of a functional GBA [196]. The functional GBA involves a complex bidirectional network encompassing the central nervous system (CNS), autonomic nervous system (ANS), ENS, vagus nerve (VN), the neuroendocrine and neuroimmune systems, the HPA, and the gut/gut microbiota. The microbiota play a crucial role in the GBA, contributing to nutrient bioconversion, detoxification, immune regulation, and protection against pathogens. Research indicates that maternal gut microbiota play a critical role in fetal development by transferring metabolites and other factors to the fetus through the placenta [197].

Maternal gut microbiota play a crucial role in supporting the healthy development of the fetal brain, influencing both its structural and functional connectivity and ultimately affecting cognitive performance and behavioral outcomes in offspring. Clinical studies indicate that disruptions in maternal gut microbiota, such as dysbiosis during pregnancy, can negatively impact fetal CNS’s physiological and functional development. Instances of microbial depletion, caused by factors like infection or antibiotic treatment during pregnancy, have been associated with abnormal brain structure and function, contributing to maladaptive behaviors reminiscent of autism in offspring [138,139]. Depletion of maternal gut microbiota during pregnancy impacts gene expression in the developing fetal brain, particularly that of genes that regulate the development of new axons connecting the thalamus to the cortex, which is responsible for sensory processes. Gut microbiota generate neurotransmitters and neuromodulators, including serotonin, gamma-aminobutyric acid (GABA), and SCFAs. These bioactive substances are transported to the fetal brain through the placenta and blood–brain barrier. Maternal gut microbiota, particularly during pregnancy, produce SCFAs crucial for various differentiations facilitated by G protein-coupled receptors.

Additionally, maternal gut metabolites support fetal thalamocortical axogenesis. Notably, germ-free mice exhibited region-specific changes in neurotransmitter systems, with increased serotonin (5-HT) levels in the hippocampus [194]. The placenta plays a crucial role in synthesizing 5-HT, influencing fetal CNS development by regulating cell proliferation, migration, and wiring during development. Tryptophan, the precursor to 5-HT, is derived from maternal gut metabolites. Placental 5-HT reaches the fetal forebrain during initial axon growth and cortical neurogenesis, with serotonergic neurons appearing in the fetal hindbrain around embryonic day 10.5 in mice [198]. Chronic mild stress during rat pregnancy increases free tryptophan levels in both maternal blood and the fetal brain, leading to heightened anxiety in offspring. In humans, placental synthesis of 5-HT occurs in the first and second trimesters of pregnancy [199]. Disruption of placental 5-HT signaling causes long-term behavioral problems, including anxiety after birth. The maternal gut microbiome also influences the formation of the fetal BBB. Animal studies have demonstrated that higher BBB permeability in germ-free mice is related to poorer innate immunity in the brain and to reduced thalamocortical axon development [34]. The VN plays a significant role in innervating the intestine from the proximal duodenum to the distal descending colon, serving as a crucial bidirectional communication pathway between the gut and the brain. It conveys information about the gut’s status, including chemical content, distension, and inflammation, to key brain regions such as the nucleus of the solitary tract (NTS), the paraventricular nucleus of the hypothalamus (PVN), and the arcuate nucleus. Metabolites produced by maternal gut microbiota, including SCFAs, are transported through specific transporters across the gut epithelium, activating the VN.

Afferent fibers of the VN carry signals from the gut microbiota to the brain. In response to these signals, the brain sends feedback signals back to entero-epithelial cells through efferent fibers of the VN. This bidirectional communication system allows for the regulation of gut–brain interactions [200]. VN sensory and motor nuclei were discovered in E11-E14 mouse embryos [201]. In humans, there is a rapid rise in myelinated vagal fibers from 24 weeks until adolescence, with the most rapid rise reported from gestational weeks 30–32 to 6 months after birth [202]. During the gestational transition from the late second to the early third trimester, sympathetic activation increases, accompanied by increased parasympathetic regulation and baseline stability ability [179]. During the final trimester, the myelination of vagal efferent fibers associated with heart activities begins [174].

**Figure 3:**
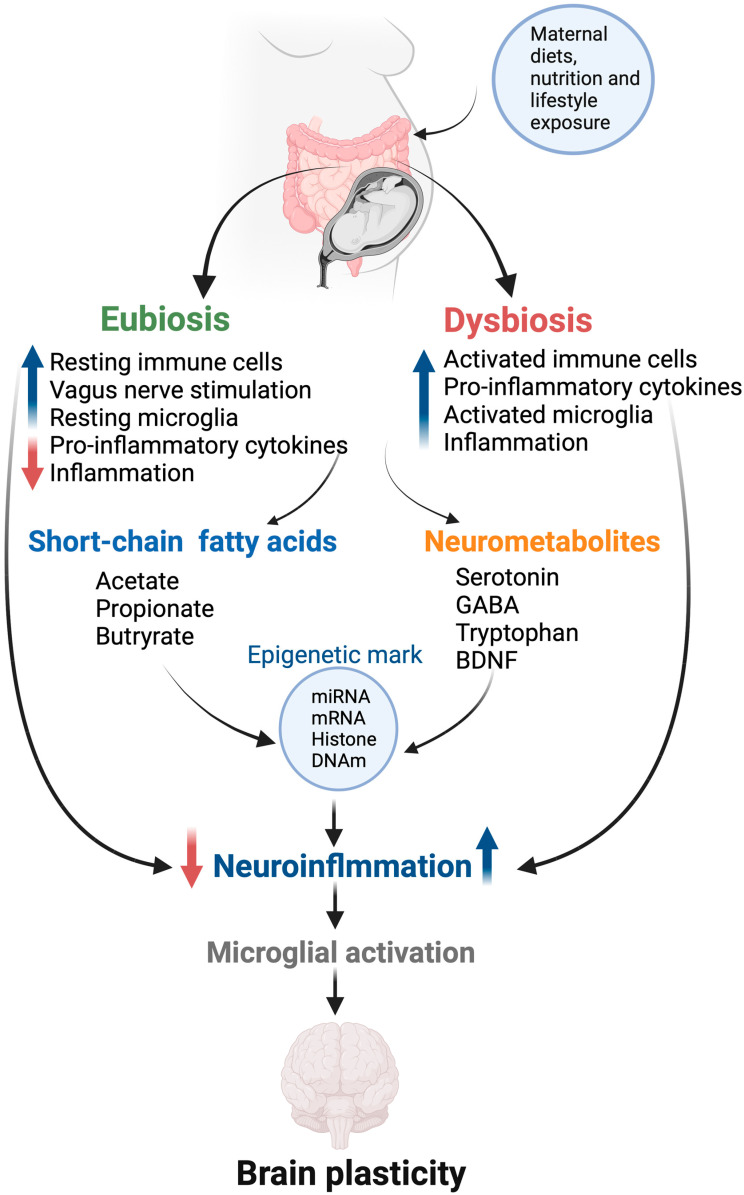
Mechanistic overview of gut microbial effects on fetal brain plasticity. Gut microflora produces several neuroactive metabolites such as GABA, tryptophan, and serotonin, potentially regulating neurotransmitter homeostasis in the fetal gut–brain axis. The gut microbiota also affect neuroinflammation and microglial maturation, and they function in a mechanism involving SCFA action and/or modulation of neuroactive molecules. These metabolites, in turn, activate the expression of epigenetic marks, including mRNA, miRNA, hydroxymethylation, and histone remodeling in the brain cells. BDNF—brain-derived neurotrophic factor; GABA—gamma-aminobutyric acid; DNAm—DNA methylation.

The gut microbiota are involved in bidirectional cross-talk with the brain through the GBA, encompassing the CNS and ANS, sympathetic and parasympathetic components, including the VN and ENS, as well as the immune, endocrine (including the hypothalamic–pituitary–adrenal or HPA), and gut/gut microbiota systems (Figure 3). The reciprocal GBA network enables communication between the brain’s cognitive and emotional centers and the ENS’s peripheral intestinal functions [203]. The gut microbiota produce hormones, metabolites, and neurotransmitters, creating a connection between the gut and the brain. Conversely, the brain influences intestinal activities, including the behavior of functional immune effector cells. It is improbable that the prenatal gut microbiota can independently generate metabolites and influence CNS development. It is reasonable to infer that maternal microbial metabolites and other factors might be involved in fetal development, including the formation of the GBA. This inference gains support from observations indicating that disruptions in maternal gut microbiota during pregnancy are linked to irregularities in the developing GBA, encompassing neuronal, gastrointestinal, immune, and hormonal components [204]. Maternal gut dysbiosis activates the microglia, leading to systemic inflammation and neuroinflammation [205]. While eubiosis promotes myelination, dysbiosis induces pro-inflammatory cytokines that can damage differentiating neurons and oligodendrocytes during active myelination, supporting the notion of maternal gut dysbiosis-induced inflammation on fetal neuroinflammation [195].

Maternal gut microbiota activate the fetal neuroendocrine HPA axis, producing cortisol and influencing the physiological stress response [206]. This activation occurs by releasing pro-inflammatory mediators, prostaglandins, and microbial antigens that can cross the BBB. Maternal gut dysbiosis can lead to constitutive hyperactivity of the HPA axis [207]. The fetal HPA axis controls the maturation of the fetal liver, lungs, brain, and other organs. A significant portion of neuroendocrine maturation occurs in utero in mammals (such as primates, sheep, and guinea pigs) to produce mature young [152]. In contrast, species that produce immature young (like rats, rabbits, and mice) exhibit predominant neuroendocrine development postnatally.

Consequently, manipulations during the fetal or neonatal stages affect different neuroendocrine developmental stages, depending on the species studied [208]. The human HPA axis becomes active at 11 weeks’ gestation, with hormonal activity detectable between 8 and 12 weeks. For example, the expression of glucocorticoid receptor (GR) mRNA was noted in the adrenal gland as early as 8–10 weeks of life, with limited knowledge about later developmental changes in GR expression [209]. Corticotropin-releasing hormone (CRH) immunoactivity and bioactivity are evident in fetal hypothalamic tissue extracts by 12–13 weeks gestation, increasing with gestational age [210]. During pregnancy, the fetal hypothalamus and the placenta produce CRH, regulating HPA axis maturation and adrenocorticotrophin (ACTH) secretion. ACTH, in turn, coordinates fetal adrenocortical growth, angiogenesis, differentiation, and steroidogenesis. Cortisol plays a crucial role in maintaining intrauterine homeostasis and fetal tissue maturation, with de novo synthesis starting in humans after 28 weeks of gestation. In response to acute stress like arterial hypotension, the fetal hypothalamus releases CRH, stimulating the secretion of fetal ACTH, which enhances cortisol production [211]. Placental estrogens can also modulate fetal cortisol levels by altering the fetal HPA axis, which involves the conversion of active cortisol to inactive cortisone or vice versa [212].

## 12. Conclusions

Epigenetic regulation maintains gestational integrity and fetoplacental development. Unveiling epigenomic modification can expand our understanding of the dynamic developmental processes to intervene for a better new life. Disseminating epigenetic impacts on developmental disease would sensitize clinicians to advise women at increased risk of adverse pregnancy; as a result, they might develop personalized, risk-specific interventions. Several clinical trials have highlighted the effects of maternal nutrients on fetal epigenetic programming, which influences fetal growth, birth weight, and brain performance in subsequent stages of life (Table 2).

Although it is widely agreed that epigenomic dysregulation can program later disease risk in the offspring, effective interventions aimed at modulating the epigenome to manage complex human diseases due to nutritional programming are obscure. Early biological markers should be established prenatally to detect traces in system pathophysiology (nonspecific tissue) or local tissue (such as the placenta). Epigenetic alterations linked to maternal nutrition and environmental exposures may affect fetal growth and development via programming. Although nutritional epigenetics has been viewed as an attractive tool for preventing pediatric developmental diseases, current knowledge is limited and warrants integrated studies to include available tools like artificial intelligence, resources such as next-generation sequencing or mass spectrometry, and other omics strategies.

## Figures and Tables

**Table 1:** Fetal epigenetic modifications due to maternal diet intervention: evidence from pre-clinical trials.

Aim	Study Design	Primary Outcome	Refs.
To distinguish between direct transmission of epigenetic states and de novo epigenetically induced marks in each generation in response to energy diets	Female Wistar rats, dietary energy increased by 25% at conception in F0 and maintained up to generation F3; epigenetic marks over four generations studied	- Transgenerational effects on offspring’s phenotype directly reflected in gene-specific DNA methylation - Induction of de novo epigenetic marks in each generation indicated heritability of epigenetic changes due to germline transmission	[42]
Effect of dietary choline on fetal brain and memory function in deficient mice	Timed-pregnant C57 BL/6 mice, choline diet—1.1 g/kg choline chloride from embryonic day 12 to day 17, brain analysis on day 17	- Decreased DNA global methylation in the ventricular and subventricular zones of the choline-deficient fetal brain - Decreased DNAm of gene (*Cdkn3*) expression of a cell cycle regulator indicated altered brain development	[14]
Restricted prenatal diet and transgenerational epigenetic alterations in four successive generations in rats	Wistar rats, control—chow diet, restricted group—50% of their daily intake from the appearance of vaginal plug until parturition	- Prenatal dietary restriction affected the expression of genes involved in epigenetic mechanisms in the liver across generations - Influenced the global histone H3 acetylation in fetal liver	[43]
n-3 PUFA deficiency at the time of pregnancy and lactation and epigenetic changes in the brain of adult offspring in mice	C57BL/6J mice, n-3 PUFA deficient diet—sunflower oil with high LA and no n-3 PUFA; n-3 PUFA adequate diet—fish oil, flax seed and sunflower oil containing 0.47% DHA and 5.25% LA	- Decreased *BDNF* expression in deficient diet associated with a hypermethylated DNA at CpG sites indicated a potential long-term impacts of brain development in offspring	[44]
Effect of folic acid availability or deficiency on oocyte development and epigenetic effects in next-generation progeny	Female BALB/c mice, deficient in (7-fold) or supplemented with (10-fold) folic acid starting from 4 wks prior to mating throughout gestation and lactation	- F2 litters showed a higher resorption rate, reduced litter size, and abnormal embryo outcomes - High or low folate before oocyte maturation affected oocyte quality and development in subsequent generation by altering DNA methylation	[45]
Maternal n-3 PUFA deficiency and changes in the epigenetic modulators of the placenta	Female Swiss albino mice, n-3 PUFA deficient diet—n-6/n-3 PUFA = 50/1, 0.13% energy from ALA; n-3 sufficient diet—n-6/n-3 PUFA = 2/1; 2.26% energy from ALA	- Global hypermethylation in F0 and F1 of n-3 deficient placenta - Increased expression of DNMT3A and DNMT3B in F0 and F1 n-3 deficient placenta	[12]
Effect of maternal micronutrients and omega-3 fatty acids on global methylation patterns in the brain	Pregnant rats fed with folic acid (normal and excess) in presence or absence of Vit B12; omega-3 supplementation in Vit B12-deficient rats	- Global hypomethylation in offspring brain at birth - Despite postnatal control diet, cortex of offspring showed hypermethylation at adult age - Omega-3 fatty acids reversed methylation patterns	[46]
Effects of dietary combinations of B12 and folic acid on genome-imprinting modulators	6-week-old C57BL/6 male mice fed with standard chow diet and females with different levels of folic acid and B12 for two generations	Non-coding RNA expression of IGF2R and KCNQ1OT1 sensitive to different dietary combinations folic acid and B12 in mouse offspring, indicating epigenetic programming	[47]
Maternal low protein diet (MLPD) and epigenetic alterations in brain renin–angiotensin system (RAS) in mice	FVB/NJ mice, MLPD—50% protein depletion, from gD10.5 to 17.5	- MLPD results in hypomethylation of ACE-1 promoter regions in fetal brain - Changes in DNA methylation and miRNA expression modulate key regulators of hypertension in adults	[48]
Effect of maternal HFD on fetal gluconeogenic gene expression and regulation of histone modifications	Timed-pregnant obese resistant rats, control—64%, 20%, 16%; HFD—35%, 20%, 45% of carbohydrates, proteins, and fats, respectively, from embryonic day 2 to 20	In utero exposure to HFD programmed the gluconeogenic capacity of offspring through epigenetic modifications and predisposed offspring with altered insulin sensitivity in adulthood	[49]
Maternal calorie restriction (CR) on DNA methylation and placental gene expression	C57/BL6 mice, control—chow diet, CR—50% (*w*/*w*) daily intake from gD 10 to 19	Altered DMRs in CR mice associated with IUGR phenotypes enriched with micro-RNA target genes linked with risk factors of cardiovascular and neurological diseases	[50]
Maternal HFD during pregnancy and its effects on regulation of gene functions	Female C57BL6J, HFD—22.6% fat, 48.6% carbohydrate, 23% protein; standard chow diet—10% fat, 68.8% carbohydrate, 18% protein; prior to conception and during pregnancy and lactation	Altered hepatic expression of insulin-like growth factor-2 and key microRNAs in adult offspring	[51]

PUFA—polyunsaturated fatty acids; LA—linoleic acid; ALA—alpha-linolenic acid; DHA—docosahexaenoic acid; HFD—high-fat diet; BDNF—brain-derived neurotrophic factor; DMR—differentially methylated regions; Cdkn3—cyclin-dependent kinase inhibitor 3; ACE-1—angiotensin-converting enzyme 1; IUGR—intrauterine growth restriction; DNMT3A—DNA methyltransferase 3A; DNMT3B—DNA methyltransferase 3B; gD—gestational day.

**Table 2:** Role of maternal nutrients in fetal epigenetic programming: consolidated clinical trials.

Aim	Subjects and Design	Analysis and Method	Primary Outcome	Refs.
In utero DHA supplement and fetal epigenome	- Pregnant women in Australia - DHA (800 mg) and EPA (100 mg) supplementation per day from gD 21 until delivery	Blood spots at birth (*n* = 991)	- Twenty-one differentially methylated regions (DMRs) in genes associated with appetite regulation and neurodevelopment - DMRs was greater in males than females	[213]
Association between maternal fatty acids (FAs) and newborn DNA methylation (DNAm)	- Pregnant women in the United States - Plasma phospholipid FAs at preconception 4 mo before pregnancy (*n* = 346) and at 8 wks (*n* = 374) of gestation	Cord blood DNA from singletons	- Preconception marine PUFA levels correlated with increased DNAm of *GRAMD2A* and *HTR1B* genes involved in neurological functions - Preconception SFA levels correlated with increased DNAm at *KIF25-AS1* and *SLC39A14* genes involved in microtubule motor activity and neurodegeneration, respectively - FAs levels at 8 weeks of gestation were unrelated to DNAm	[214]
Dietary PUFAs during pregnancy and methylation of imprinted genes	DHA (400 mg/day, *n* = 131) or placebo (*n* = 130) from gD 18–22 until delivery	DNA methylation of IGF2 promoter 3 (P3), IGF2 DMRs, and H19 DMR in cord blood mononuclear cells	- DNAm of *IGF2 P3* higher (*p* = 0.04) in preterm infants of DHA group than placebo - *IGF2* DMRs higher in DHA group than placebo infants of overweight mothers - *H19* DMR lower in infants of normal-weight mothers in DHA group	[108]
Maternal micronutrient supplementation and DNAm in their children	Indian (*n* = 698) and Gambian (*n* = 293) pregnant women before and during pregnancy	- Micronutrient-rich snack periconceptionally (India) - Micronutrient tablet daily until confirmation of pregnancy (Gambia)	- No changes in DNAm children (5–7 yrs) from the Indian cohort - In the Gambian cohort, changes in DMRs involved in angiogenesis and cell growth; cadherin 18 (*CDH18*) implicated in brain development	[215]
Prenatal n-3 PUFA intake and DMRs in cord blood	Pregnant women, *n* = 577, Italy	Pregnant women: 45–64% of daily calories from carbohydrates, 20–35% of daily calories from fats, and 60 g/day of proteins; 3 portions of fish per week	- Reduced methylation of *MSTN*, *ATP8B3*, *IFNA13*, and *GABBR2* genes in newborns from either low- or high-n-3 PUFA diet compared to medium-n-3 PUFA diet - Overexpression of these genes may lead to insulin resistance and adiposity	[216]
Relationship of maternal dietary FA quality with epigenetic aging and newborn cardiometabolic risk	Healthy mothers and children, *n* = 224, Australia	Body fat, aortic intima media thickness, heart rate variability, and epigenetic age acceleration in newborn infants	- Omega-3 FAs showed a beneficial association with newborn epigenetic age acceleration - Maternal dietary FA quality associated with epigenetic aging in newborns	[217]
Vitamin D supplementation and epigenetic gestational age acceleration (GAA) in newborns	Pregnant women, *n* = 92, multiethnic population, USA	- 4000 IU/day Vit D3 or placebo plus prenatal 400 IU vitamin D3 during pregnancy	- GAA associated with higher birth weight - No association between vitamin D3 supplementation and GAA	[218]
Folic acid beyond first trimester and DNAm of genes related to brain development and function	Pregnant women, *n* = 86, Ireland	400 µg folic acid/day through the second and third trimesters compared with placebo	- Lower DNAm of *LINE-1*, *IGF2*, and *BDNF* - Continued folic acid supplementation in pregnancy results in significant changes in DNAm of genes related to brain development	[219]
Effect of maternal choline intake on methylations of cortisol regulating genes in placenta and cord venous blood	Pregnant women, *n* = 26, USA	Choline supplementation: 480 mg/day and 930 mg/day for 12 weeks from 26–29 gestational weeks until delivery	- Increased promoter methylation of cortisol regulating *CRH* and *NR3C1* genes - Maternal choline alters the epigenetic state of genes that regulate fetal HPA axis	[16]
Effect of maternal dietary glycemic index on cord blood DNAm	Mother–offspring pairs, *n* = 2003, U.K., Netherlands and Spain	Maternal dietary glycemic index calculated by FFQ and correlated with cord blood DNAm	Maternal glycemic index associated with changes in DNAm of genes associated with neurodevelopment and lipid metabolism in overweight/obesity subjects	[220]
Parental dietary quality on offspring DNAm	Families, *n* = 1124, Ireland	Maternal diet in first trimester and paternal diet were assessed by FFQ	- Maternal healthy eating index inversely associated with DNAm of *PLEKHM1* gene (bone development) - Prenatal parental dietary quality might influence offspring DNAm during childhood	[221]
Mediterranean diet in pregnancy and neonatal DNA methylation at birth	Mother–infant pairs, *n*= 390, USA	Overall dietary assessment by FFQ in periconception and once in each trimester	- Lower adherence to diet results in a greater odd of hypomethylation at the MEG3-IG DMR indicating dietary effects on genome imprinting	[222]
Mediterranean diet adherence during pregnancy and imprinted gene methylation of brain task	Mother–infant pairs, *n* = 325, USA	Maternal periconceptional FFQ and correlated with child behavioral outcomes at 2 years of age	- Adherence to a Mediterranean diet in early pregnancy was associated with favorable neurobehavioral outcomes in early childhood - Sex-dependent methylation differences of *MEG3* and *IGF2 DMRs*	[223]
Dietary fat intake during pregnancy and imprinting gene methylations	Pregnant women, *n* = 154, Eastern Massachusetts	Maternal fat intake during first and second trimester by FFQ	Maternal total fat and PUFA intake inversely correlated with *IGF2-DMR* and positively correlated with *H19-DMR* methylation	[224]
Effect of substituted low-glycemic-index (GI) diet or GI diet on epigenetic profile of offspring at 5 years of age	5-year-old children from ROLO kids’ study, *n* = 60–63, Australia, Ireland	High-GI diet substituted with low-GI from second trimester until delivery	No association between maternal factors due to substituted GI or low-GI diet on DNAm status of offspring at 5 years of age	[225,226]
Mediterranean diet adherence during pregnancy, fetal gut microbiota and offspring epigenetic regulation	Pregnant women, *n* = 41, USA	Dietary patterns assessed by FFQ, Mediterranean diet adherence scores correlated with neonatal microbiome and fetal epigenetic programming	Adherence to Mediterranean diet results in a greater abundance of *Pasteurellaceae*, *Bacteroidaceae*, and other short-chain fatty acid-producing species and is connected with fetal DMRs of in utero development	[227]

SFA—saturated fatty acid; PUFA—polyunsaturated fatty acid; EPA—eicosapentaenoic acid; DHA—docosahexaenoic acid; HPA—hypothalamic–pituitary–adrenal axis; DMR—differentially methylated regions; DNAm—DNA methylation; GRAMD2A—GRAM domain containing 2A; HTR1B—5-Hydroxytryptamine Receptor 1B; KIF25-AS1—Kinesin Family Member 25 Antisense RNA 1; SLC39A14—solute carrier family 39 member 14; IGF2—insulin-like growth factor 2; H19—gene for a long non-coding RNA; MSTN—myostatin; ATP8B3—ATPase Phospholipid Transporting 8B3; IFNA13—Interferon Alpha 13; GABBR2—gamma-aminobutyric acid type B receptor subunit 2; FFQ—food frequency questionnaire; LINE-1—long interspersed element 1; BDNF—brain-derived neurotrophic factor; CRH—cortisol-regulating hormone; NR3C1—glucocorticoid receptor; PLEKHM1—pleckstrin homology and RUN domain containing M1; MEG3—maternally expressed 3 gene; gD—gestational day.

## References

[1] Duttaroy A.K. (2016). Docosahexaenoic acid supports feto-placental growth and protects cardiovascular and cognitive function: A mini review. Eur. J. Lipid Sci. Technol..

[2] Dimasuay K.G., Boeuf P., Powell T.L., Jansson T. (2016). Placental Responses to Changes in the Maternal Environment Determine Fetal Growth. Front. Physiol..

[3] Duttaroy A.K. (2023). Influence of Maternal Diet and Environmental Factors on Fetal Development. Nutrients.

[4] Duttaroy A.K. (2009). Transport of fatty acids across the human placenta: A review. Prog. Lipid Res..

[5] Basak S., Duttaroy A.K. (2023). Maternal PUFAs, Placental Epigenetics, and Their Relevance to Fetal Growth and Brain Development. Reprod. Sci..

[6] Kuzawa C.W. (2005). Fetal origins of developmental plasticity: Are fetal cues reliable predictors of future nutritional environments?. Am. J. Hum. Biol..

[7] Bird A. (2007). Perceptions of epigenetics. Nature.

[8] Chandak G.R., Silver M.J., Saffari A., Lillycrop K.A., Shrestha S., Sahariah S.A., Di Gravio C., Goldberg G., Tomar A.S., Betts M. (2017). Protocol for the EMPHASIS study; epigenetic mechanisms linking maternal pre-conceptional nutrition and children’s health in India and Sub-Saharan Africa. BMC Nutr..

[9] Yajnik C.S., Deshpande S.S., Jackson A.A., Refsum H., Rao S., Fisher D.J., Bhat D.S., Naik S.S., Coyaji K.J., Joglekar C.V. (2008). Vitamin B12 and folate concentrations during pregnancy and insulin resistance in the offspring: The Pune Maternal Nutrition Study. Diabetologia.

[10] Burdge G.C., Hanson M.A., Slater-Jefferies J.L., Lillycrop K.A. (2007). Epigenetic regulation of transcription: A mechanism for inducing variations in phenotype (fetal programming) by differences in nutrition during early life?. Br. J. Nutr..

[11] Burdge G.C., Lillycrop K.A. (2014). Fatty acids and epigenetics. Curr. Opin. Clin. Nutr. Metab. Care.

[12] Srinivas V., Molangiri A., Mallepogu A., Kona S.R., Ibrahim A., Duttaroy A.K., Basak S. (2021). Maternal n-3 PUFA deficiency alters uterine artery remodeling and placental epigenome in the mice. J. Nutr. Biochem..

[13] Srinivas V., Varma S., Kona S.R., Ibrahim A., Duttaroy A.K., Basak S. (2023). Dietary omega-3 fatty acid deficiency from pre-pregnancy to lactation affects expression of genes involved in hippocampal neurogenesis of the offspring. Prostaglandins Leukot. Essent. Fat. Acids.

[14] Niculescu M.D., Craciunescu C.N., Zeisel S.H. (2006). Dietary choline deficiency alters global and gene-specific DNA methylation in the developing hippocampus of mouse fetal brains. FASEB J..

[15] Craciunescu C.N., Albright C.D., Mar M.H., Song J., Zeisel S.H. (2003). Choline availability during embryonic development alters progenitor cell mitosis in developing mouse hippocampus. J. Nutr..

[16] Jiang X., Yan J., West A.A., Perry C.A., Malysheva O.V., Devapatla S., Pressman E., Vermeylen F., Caudill M.A. (2012). Maternal choline intake alters the epigenetic state of fetal cortisol-regulating genes in humans. FASEB J..

[17] Smith Z.D., Chan M.M., Humm K.C., Karnik R., Mekhoubad S., Regev A., Eggan K., Meissner A. (2014). DNA methylation dynamics of the human preimplantation embryo. Nature.

[18] Okae H., Chiba H., Hiura H., Hamada H., Sato A., Utsunomiya T., Kikuchi H., Yoshida H., Tanaka A., Suyama M. (2014). Genome-wide analysis of DNA methylation dynamics during early human development. PLoS Genet..

[19] Slieker R.C., Bos S.D., Goeman J.J., Bovee J.V., Talens R.P., van der Breggen R., Suchiman H.E., Lameijer E.W., Putter H., van den Akker E.B. (2013). Identification and systematic annotation of tissue-specific differentially methylated regions using the Illumina 450k array. Epigenetics Chromatin.

[20] Slieker R.C., Roost M.S., van Iperen L., Suchiman H.E., Tobi E.W., Carlotti F., de Koning E.J., Slagboom P.E., Heijmans B.T., Chuva de Sousa Lopes S.M. (2015). DNA Methylation Landscapes of Human Fetal Development. PLoS Genet..

[21] Faulk C., Dolinoy D.C. (2011). Timing is everything: The when and how of environmentally induced changes in the epigenome of animals. Epigenetics.

[22] Fukumoto K., Ito K., Saer B., Taylor G., Ye S., Yamano M., Toriba Y., Hayes A., Okamura H., Fustin J.-M. (2022). Excess S-adenosylmethionine inhibits methylation via catabolism to adenine. Commun. Biol..

[23] Inoue S., Honma K., Mochizuki K., Goda T. (2015). Induction of histone H3K4 methylation at the promoter, enhancer, and transcribed regions of the Si and Sglt1 genes in rat jejunum in response to a high-starch/low-fat diet. Nutrition.

[24] Fang M., Chen D., Yang C.S. (2007). Dietary polyphenols may affect DNA methylation. J. Nutr..

[25] Dolinoy D.C., Weidman J.R., Waterland R.A., Jirtle R.L. (2006). Maternal genistein alters coat color and protects Avy mouse offspring from obesity by modifying the fetal epigenome. Environ. Health Perspect..

[26] Day J.K., Bauer A.M., DesBordes C., Zhuang Y., Kim B.E., Newton L.G., Nehra V., Forsee K.M., MacDonald R.S., Besch-Williford C. (2002). Genistein alters methylation patterns in mice. J. Nutr..

[27] Cooper W.N., Khulan B., Owens S., Elks C.E., Seidel V., Prentice A.M., Belteki G., Ong K.K., Affara N.A., Constancia M. (2012). DNA methylation profiling at imprinted loci after periconceptional micronutrient supplementation in humans: Results of a pilot randomized controlled trial. FASEB J..

[28] Li C.C., Cropley J.E., Cowley M.J., Preiss T., Martin D.I., Suter C.M. (2011). A sustained dietary change increases epigenetic variation in isogenic mice. PLoS Genet..

[29] Li Y. (2018). Epigenetic Mechanisms Link Maternal Diets and Gut Microbiome to Obesity in the Offspring. Front. Genet..

[30] Ruiz-Trivino J., Alvarez D., Cadavid J.A., Alvarez A.M. (2023). From gut to placenta: Understanding how the maternal microbiome models life-long conditions. Front. Endocrinol..

[31] Basak S., Das R.K., Banerjee A., Paul S., Pathak S., Duttaroy A.K. (2022). Maternal Obesity and Gut Microbiota Are Associated with Fetal Brain Development. Nutrients.

[32] Cui J., Wang J., Wang Y. (2023). The role of short-chain fatty acids produced by gut microbiota in the regulation of pre-eclampsia onset. Front. Cell. Infect. Microbiol..

[33] Nakajima A., Habu S., Kasai M., Okumura K., Ishikawa D., Shibuya T., Kobayashi O., Osada T., Ohkusa T., Watanabe S. (2020). Impact of maternal dietary gut microbial metabolites on an offspring’s systemic immune response in mouse models. Biosci. Microbiota Food Health.

[34] Braniste V., Al-Asmakh M., Kowal C., Anuar F., Abbaspour A., Toth M., Korecka A., Bakocevic N., Ng L.G., Kundu P. (2014). The gut microbiota influences blood-brain barrier permeability in mice. Sci. Transl. Med..

[35] Miko E., Csaszar A., Bodis J., Kovacs K. (2022). The Maternal-Fetal Gut Microbiota Axis: Physiological Changes, Dietary Influence, and Modulation Possibilities. Life.

[36] Millership S.J., Van de Pette M., Withers D.J. (2019). Genomic imprinting and its effects on postnatal growth and adult metabolism. Cell. Mol. Life Sci..

[37] Anway M.D., Cupp A.S., Uzumcu M., Skinner M.K. (2005). Epigenetic transgenerational actions of endocrine disruptors and male fertility. Science.

[38] Cropley J.E., Suter C.M., Beckman K.B., Martin D.I. (2006). Germ-line epigenetic modification of the murine A vy allele by nutritional supplementation. Proc. Natl. Acad. Sci. USA.

[39] Rakyan V.K., Beck S. (2006). Epigenetic variation and inheritance in mammals. Curr. Opin. Genet. Dev..

[40] Ng S.F., Lin R.C., Laybutt D.R., Barres R., Owens J.A., Morris M.J. (2010). Chronic high-fat diet in fathers programs beta-cell dysfunction in female rat offspring. Nature.

[41] Lesch B.J., Tothova Z., Morgan E.A., Liao Z., Bronson R.T., Ebert B.L., Page D.C. (2019). Intergenerational epigenetic inheritance of cancer susceptibility in mammals. eLife.

[42] Burdge G.C., Hoile S.P., Uller T., Thomas N.A., Gluckman P.D., Hanson M.A., Lillycrop K.A. (2011). Progressive, Transgenerational Changes in Offspring Phenotype and Epigenotype following Nutritional Transition. PLoS ONE.

[43] Nowacka-Woszuk J., Szczerbal I., Malinowska A.M., Chmurzynska A. (2018). Transgenerational effects of prenatal restricted diet on gene expression and histone modifications in the rat. PLoS ONE.

[44] Fan C., Fu H., Dong H., Lu Y., Lu Y., Qi K. (2016). Maternal n-3 polyunsaturated fatty acid deprivation during pregnancy and lactation affects neurogenesis and apoptosis in adult offspring: Associated with DNA methylation of brain-derived neurotrophic factor transcripts. Nutr. Res..

[45] Ly L., Chan D., Landry M., Angle C., Martel J., Trasler J. (2020). Impact of mothers’ early life exposure to low or high folate on progeny outcome and DNA methylation patterns. Environ. Epigenetics.

[46] Sable P., Randhir K., Kale A., Chavan-Gautam P., Joshi S. (2015). Maternal micronutrients and brain global methylation patterns in the offspring. Nutr. Neurosci..

[47] Mahajan A., Sapehia D., Bagga R., Kaur J. (2021). Different dietary combinations of folic acid and vitamin B12 in parental diet results in epigenetic reprogramming of IGF2R and KCNQ1OT1 in placenta and fetal tissues in mice. Mol. Reprod. Dev..

[48] Goyal R., Goyal D., Leitzke A., Gheorghe C.P., Longo L.D. (2010). Brain Renin-Angiotensin System: Fetal Epigenetic Programming by Maternal Protein Restriction during Pregnancy. Reprod. Sci..

[49] Strakovsky R.S., Zhang X., Zhou D., Pan Y.-X. (2011). Gestational high fat diet programs hepatic phosphoenolpyruvate carboxykinase gene expression and histone modification in neonatal offspring rats. J. Physiol..

[50] Chen P.-Y., Ganguly A., Rubbi L., Orozco L.D., Morselli M., Ashraf D., Jaroszewicz A., Feng S., Jacobsen S.E., Nakano A. (2013). Intrauterine calorie restriction affects placental DNA methylation and gene expression. Physiol. Genom..

[51] Zhang J., Zhang F., Didelot X., Bruce K.D., Cagampang F.R., Vatish M., Hanson M., Lehnert H., Ceriello A., Byrne C.D. (2009). Maternal high fat diet during pregnancy and lactation alters hepatic expression of insulin like growth factor-2 and key microRNAs in the adult offspring. BMC Genom..

[52] Maloyan A., Muralimanoharan S., Huffman S., Cox L.A., Nathanielsz P.W., Myatt L., Nijland M.J. (2013). Identification and comparative analyses of myocardial miRNAs involved in the fetal response to maternal obesity. Physiol. Genom..

[53] Wehbe N., Nasser S.A., Pintus G., Badran A., Eid A.H., Baydoun E. (2019). MicroRNAs in Cardiac Hypertrophy. Int. J. Mol. Sci..

[54] Colpaert R.M.W., Calore M. (2019). MicroRNAs in Cardiac Diseases. Cells.

[55] Duisters R.F., Tijsen A.J., Schroen B., Leenders J.J., Lentink V., van der Made I., Herias V., van Leeuwen R.E., Schellings M.W., Barenbrug P. (2009). miR-133 and miR-30 regulate connective tissue growth factor: Implications for a role of microRNAs in myocardial matrix remodeling. Circ. Res..

[56] Khorram O., Chuang T.D., Pearce W.J. (2015). Long-term effects of maternal undernutrition on offspring carotid artery remodeling: Role of miR-29c. J. Dev. Orig. Health Dis..

[57] Plagemann A. (2004). ‘Fetal programming’ and ‘functional teratogenesis’: On epigenetic mechanisms and prevention of perinatally acquired lasting health risks. J. Perinat. Med..

[58] Godfrey K.M., Lillycrop K.A., Burdge G.C., Gluckman P.D., Hanson M.A. (2007). Epigenetic mechanisms and the mismatch concept of the developmental origins of health and disease. Pediatr. Res..

[59] Sookoian S., Gianotti T.F., Burgueno A.L., Pirola C.J. (2013). Fetal metabolic programming and epigenetic modifications: A systems biology approach. Pediatr. Res..

[60] Dunlop A.L., Mulle J.G., Ferranti E.P., Edwards S., Dunn A.B., Corwin E.J. (2015). Maternal Microbiome and Pregnancy Outcomes That Impact Infant Health: A Review. Adv. Neonatal. Care.

[61] Enstad S., Cheema S., Thomas R., Fichorova R.N., Martin C.R., O’Tierney-Ginn P., Wagner C.L., Sen S. (2021). The impact of maternal obesity and breast milk inflammation on developmental programming of infant growth. Eur. J. Clin. Nutr..

[62] Calabuig-Navarro V., Puchowicz M., Glazebrook P., Haghiac M., Minium J., Catalano P., Hauguel deMouzon S., O’Tierney-Ginn P. (2016). Effect of omega-3 supplementation on placental lipid metabolism in overweight and obese women. Am. J. Clin. Nutr..

[63] Khanal P., Duttaroy A.K. (2022). Prospect of potential intrauterine programming impacts associated with COVID-19. Front. Public Health.

[64] Lassance L., Haghiac M., Leahy P., Basu S., Minium J., Zhou J., Reider M., Catalano P.M., Hauguel-de Mouzon S. (2015). Identification of early transcriptome signatures in placenta exposed to insulin and obesity. Am. J. Obstet. Gynecol..

[65] Apicella C., Ruano C.S.M., Mehats C., Miralles F., Vaiman D. (2019). The Role of Epigenetics in Placental Development and the Etiology of Preeclampsia. Int. J. Mol. Sci..

[66] Troncoso F., Acurio J., Herlitz K., Aguayo C., Bertoglia P., Guzman-Gutierrez E., Loyola M., Gonzalez M., Rezgaoui M., Desoye G. (2017). Gestational diabetes mellitus is associated with increased pro-migratory activation of vascular endothelial growth factor receptor 2 and reduced expression of vascular endothelial growth factor receptor 1. PLoS ONE.

[67] Devarshi P.P., Grant R.W., Ikonte C.J., Hazels Mitmesser S. (2019). Maternal Omega-3 Nutrition, Placental Transfer and Fetal Brain Development in Gestational Diabetes and Preeclampsia. Nutrients.

[68] Basak S., Vilasagaram S., Duttaroy A.K. (2020). Maternal dietary deficiency of n-3 fatty acids affects metabolic and epigenetic phenotypes of the developing fetus. Prostaglandins Leukot. Essent. Fat. Acids.

[69] Johnsen G.M., Basak S., Weedon-Fekjaer M.S., Staff A.C., Duttaroy A.K. (2011). Docosahexaenoic acid stimulates tube formation in first trimester trophoblast cells, HTR8/SVneo. Placenta.

[70] Duttaroy A.K., Basak S. (2022). Maternal Fatty Acid Metabolism in Pregnancy and Its Consequences in the Feto-Placental Development. Front. Physiol..

[71] Basak S., Das M.K., Duttaroy A.K. (2013). Fatty acid-induced angiogenesis in first trimester placental trophoblast cells: Possible roles of cellular fatty acid-binding proteins. Life Sci..

[72] Stuart T.J., O’Neill K., Condon D., Sasson I., Sen P., Xia Y., Simmons R.A. (2018). Diet-induced obesity alters the maternal metabolome and early placenta transcriptome and decreases placenta vascularity in the mouse. Biol. Reprod..

[73] Mohammed S., Qadri S.S.Y., Mir I.A., Kondapalli N.B., Basak S., Rajkumar H. (2022). Fructooligosaccharide ameliorates high-fat induced intrauterine inflammation and improves lipid profile in the hamster offspring. J. Nutr. Biochem..

[74] Song L., Sun B., Boersma G.J., Cordner Z.A., Yan J., Moran T.H., Tamashiro K.L.K. (2017). Prenatal high-fat diet alters placental morphology, nutrient transporter expression, and mtorc1 signaling in rat. Obesity.

[75] Saben J., Lindsey F., Zhong Y., Thakali K., Badger T.M., Andres A., Gomez-Acevedo H., Shankar K. (2014). Maternal obesity is associated with a lipotoxic placental environment. Placenta.

[76] Shrestha D., Workalemahu T., Tekola-Ayele F. (2019). Maternal dyslipidemia during early pregnancy and epigenetic ageing of the placenta. Epigenetics.

[77] Dube E., Gravel A., Martin C., Desparois G., Moussa I., Ethier-Chiasson M., Forest J.C., Giguere Y., Masse A., Lafond J. (2012). Modulation of fatty acid transport and metabolism by maternal obesity in the human full-term placenta. Biol. Reprod..

[78] Segura M.T., Demmelmair H., Krauss-Etschmann S., Nathan P., Dehmel S., Padilla M.C., Rueda R., Koletzko B., Campoy C. (2017). Maternal BMI and gestational diabetes alter placental lipid transporters and fatty acid composition. Placenta.

[79] Dutta-Roy A.K. (2000). Transport mechanisms for long-chain polyunsaturated fatty acids in the human placenta. Am. J. Clin. Nutr..

[80] Gazquez A., Prieto-Sanchez M.T., Blanco-Carnero J.E., Ruiz-Palacios M., Nieto A., van Harskamp D., Oosterink J.E., Schierbeek H., van Goudoever J.B., Demmelmair H. (2020). Altered materno-fetal transfer of 13C-polyunsaturated fatty acids in obese pregnant women. Clin. Nutr..

[81] Hirschmugl B., Desoye G., Catalano P., Klymiuk I., Scharnagl H., Payr S., Kitzinger E., Schliefsteiner C., Lang U., Wadsack C. (2017). Maternal obesity modulates intracellular lipid turnover in the human term placenta. Int. J. Obes..

[82] Laskewitz A., van Benthem K.L., Kieffer T.E.C., Faas M.M., Verkaik-Schakel R.N., Plosch T., Scherjon S.A., Prins J.R. (2019). The influence of maternal obesity on macrophage subsets in the human decidua. Cell. Immunol..

[83] Nogues P., Dos Santos E., Jammes H., Berveiller P., Arnould L., Vialard F., Dieudonne M.N. (2019). Maternal obesity influences expression and DNA methylation of the adiponectin and leptin systems in human third-trimester placenta. Clin. Epigenetics.

[84] Gauster M., Hiden U., van Poppel M., Frank S., Wadsack C., Hauguel-de Mouzon S., Desoye G. (2011). Dysregulation of placental endothelial lipase in obese women with gestational diabetes mellitus. Diabetes.

[85] Herrera E., Ortega-Senovilla H. (2014). Lipid metabolism during pregnancy and its implications for fetal growth. Curr. Pharm. Biotechnol..

[86] Davis C.D., Ross S.A. (2007). Dietary components impact histone modifications and cancer risk. Nutr. Rev..

[87] Jansson N., Rosario F.J., Gaccioli F., Lager S., Jones H.N., Roos S., Jansson T., Powell T.L. (2013). Activation of placental mTOR signaling and amino acid transporters in obese women giving birth to large babies. J. Clin. Endocrinol. Metab..

[88] Hao M., Zhao W., Zhang L., Wang H., Yang X. (2016). Low folate levels are associated with methylation-mediated transcriptional repression of miR-203 and miR-375 during cervical carcinogenesis. Oncol. Lett..

[89] Zheng J., Zhang Q., Mul J.D., Yu M., Xu J., Qi C., Wang T., Xiao X. (2016). Maternal high-calorie diet is associated with altered hepatic microRNA expression and impaired metabolic health in offspring at weaning age. Endocrine.

[90] Cao C., Zhang H., Zhao L., Zhou L., Zhang M., Xu H., Han X., Li G., Yang X., Jiang Y. (2016). miR-125b targets DNMT3b and mediates p53 DNA methylation involving in the vascular smooth muscle cells proliferation induced by homocysteine. Exp. Cell Res..

[91] Koturbash I., Melnyk S., James S.J., Beland F.A., Pogribny I.P. (2013). Role of epigenetic and miR-22 and miR-29b alterations in the downregulation of Mat1a and Mthfr genes in early preneoplastic livers in rats induced by 2-acetylaminofluorene. Mol. Carcinog..

[92] Li X., Wu Y., Liu A., Tang X. (2016). MiR-27b is epigenetically downregulated in tamoxifen resistant breast cancer cells due to promoter methylation and regulates tamoxifen sensitivity by targeting HMGB3. Biochem. Biophys. Res. Commun..

[93] Wilting S.M., Miok V., Jaspers A., Boon D., Sorgard H., Lando M., Snoek B.C., van Wieringen W.N., Meijer C.J., Lyng H. (2016). Aberrant methylation-mediated silencing of microRNAs contributes to HPV-induced anchorage independence. Oncotarget.

[94] Du J., Cheng X., Shen L., Tan Z., Luo J., Wu X., Liu C., Yang Q., Jiang Y., Tang G. (2016). Methylation of miR-145a-5p promoter mediates adipocytes differentiation. Biochem. Biophys. Res. Commun..

[95] Tekola-Ayele F., Zeng X., Ouidir M., Workalemahu T., Zhang C., Delahaye F., Wapner R. (2020). DNA methylation loci in placenta associated with birthweight and expression of genes relevant for early development and adult diseases. Clin. Epigenetics.

[96] Sun L., Sun S. (2019). Within-sample co-methylation patterns in normal tissues. BioData Min..

[97] Clark J., Avula V., Ring C., Eaves L.A., Howard T., Santos H.P., Smeester L., Bangma J.T., O’Shea T.M., Fry R.C. (2021). Comparing the Predictivity of Human Placental Gene, microRNA, and CpG Methylation Signatures in Relation to Perinatal Outcomes. Toxicol. Sci..

[98] Agarwal P., Morriseau T.S., Kereliuk S.M., Doucette C.A., Wicklow B.A., Dolinsky V.W. (2018). Maternal obesity, diabetes during pregnancy and epigenetic mechanisms that influence the developmental origins of cardiometabolic disease in the offspring. Crit. Rev. Clin. Lab. Sci..

[99] Sinha T., Brushett S., Prins J., Zhernakova A. (2023). The maternal gut microbiome during pregnancy and its role in maternal and infant health. Curr. Opin. Microbiol..

[100] McGovern N., Shin A., Low G., Low D., Duan K., Yao L.J., Msallam R., Low I., Shadan N.B., Sumatoh H.R. (2017). Human fetal dendritic cells promote prenatal T-cell immune suppression through arginase-2. Nature.

[101] Innis S.M. (1991). Essential fatty acids in growth and development. Prog. Lipid Res..

[102] Basak S., Mallick R., Banerjee A., Pathak S., Duttaroy A.K. (2021). Maternal Supply of Both Arachidonic and Docosahexaenoic Acids Is Required for Optimal Neurodevelopment. Nutrients.

[103] Basak S., Mallick R., Duttaroy A.K. (2020). Maternal Docosahexaenoic Acid Status during Pregnancy and Its Impact on Infant Neurodevelopment. Nutrients.

[104] Crabtree J.T., Gordon M.J., Campbell F.M., Dutta-Roy A.K. (1998). Differential distribution and metabolism of arachidonic acid and docosahexaenoic acid by human placental choriocarcinoma (BeWo) cells. Mol. Cell. Biochem..

[105] Srinivas V., Molangiri A., Varma S., Mallepogu A., Kona S.R., Ibrahim A., Duttaroy A.K., Basak S. (2023). Maternal omega-3 fatty acid deficiency affects fetal thermogenic development and postnatal musculoskeletal growth in mice. J. Nutr. Biochem..

[106] DeCapo M., Thompson J.R., Dunn G., Sullivan E.L. (2019). Perinatal Nutrition and Programmed Risk for Neuropsychiatric Disorders: A Focus on Animal Models. Biol. Psychiatry.

[107] Moreno-Mendez E., Quintero-Fabian S., Fernandez-Mejia C., Lazo-de-la-Vega-Monroy M.L. (2020). Early-life programming of adipose tissue. Nutr. Res. Rev..

[108] Lee H.-S., Barraza-Villarreal A., Biessy C., Duarte-Salles T., Sly P.D., Ramakrishnan U., Rivera J., Herceg Z., Romieu I. (2014). Dietary supplementation with polyunsaturated fatty acid during pregnancy modulates DNA methylation at IGF2/H19 imprinted genes and growth of infants. Physiol. Genom..

[109] Lee H.S., Barraza-Villarreal A., Hernandez-Vargas H., Sly P.D., Biessy C., Ramakrishnan U., Romieu I., Herceg Z. (2013). Modulation of DNA methylation states and infant immune system by dietary supplementation with omega-3 PUFA during pregnancy in an intervention study. Am. J. Clin. Nutr..

[110] Refsum H., Yajnik C.S., Gadkari M., Schneede J., Vollset S.E., Orning L., Guttormsen A.B., Joglekar A., Sayyad M.G., Ulvik A. (2001). Hyperhomocysteinemia and elevated methylmalonic acid indicate a high prevalence of cobalamin deficiency in Asian Indians. Am. J. Clin. Nutr..

[111] Fryer A.A., Nafee T.M., Ismail K.M., Carroll W.D., Emes R.D., Farrell W.E. (2009). LINE-1 DNA methylation is inversely correlated with cord plasma homocysteine in man: A preliminary study. Epigenetics.

[112] Haggarty P., Hoad G., Campbell D.M., Horgan G.W., Piyathilake C., McNeill G. (2013). Folate in pregnancy and imprinted gene and repeat element methylation in the offspring. Am. J. Clin. Nutr..

[113] Chen G., Broseus J., Hergalant S., Donnart A., Chevalier C., Bolanos-Jimenez F., Gueant J.L., Houlgatte R. (2015). Identification of master genes involved in liver key functions through transcriptomics and epigenomics of methyl donor deficiency in rat: Relevance to nonalcoholic liver disease. Mol. Nutr. Food Res..

[114] Gueant J.L., Namour F., Gueant-Rodriguez R.M., Daval J.L. (2013). Folate and fetal programming: A play in epigenomics?. Trends Endocrinol. Metab..

[115] Mehedint M.G., Craciunescu C.N., Zeisel S.H. (2010). Maternal dietary choline deficiency alters angiogenesis in fetal mouse hippocampus. Proc. Natl. Acad. Sci. USA.

[116] Kovacheva V.P., Mellott T.J., Davison J.M., Wagner N., Lopez-Coviella I., Schnitzler A.C., Blusztajn J.K. (2007). Gestational choline deficiency causes global and Igf2 gene DNA hypermethylation by up-regulation of Dnmt1 expression. J. Biol. Chem..

[117] Medici V., Shibata N.M., Kharbanda K.K., Islam M.S., Keen C.L., Kim K., Tillman B., French S.W., Halsted C.H., LaSalle J.M. (2014). Maternal choline modifies fetal liver copper, gene expression, DNA methylation, and neonatal growth in the tx-j mouse model of Wilson disease. Epigenetics.

[118] Aon M.A., Cortassa S., Juhaszova M., Sollott S.J. (2016). Mitochondrial health, the epigenome and healthspan. Clin. Sci..

[119] Pauwels S., Duca R.C., Devlieger R., Freson K., Straetmans D., Van Herck E., Huybrechts I., Koppen G., Godderis L. (2016). Maternal Methyl-Group Donor Intake and Global DNA (Hydroxy)Methylation before and during Pregnancy. Nutrients.

[120] Hollis B.W., Wagner C.L. (2017). Vitamin D supplementation during pregnancy: Improvements in birth outcomes and complications through direct genomic alteration. Mol. Cell. Endocrinol..

[121] Sablok A., Batra A., Thariani K., Batra A., Bharti R., Aggarwal A.R., Kabi B.C., Chellani H. (2015). Supplementation of vitamin D in pregnancy and its correlation with feto-maternal outcome. Clin. Endocrinol..

[122] Smolders J., Menheere P., Kessels A., Damoiseaux J., Hupperts R. (2008). Association of vitamin D metabolite levels with relapse rate and disability in multiple sclerosis. Mult. Scler..

[123] Fetahu I.S., Hobaus J., Kallay E. (2014). Vitamin D and the epigenome. Front. Physiol..

[124] Dominguez-Salas P., Moore S.E., Baker M.S., Bergen A.W., Cox S.E., Dyer R.A., Fulford A.J., Guan Y., Laritsky E., Silver M.J. (2014). Maternal nutrition at conception modulates DNA methylation of human metastable epialleles. Nat. Commun..

[125] Xue J., Schoenrock S.A., Valdar W., Tarantino L.M., Ideraabdullah F.Y. (2016). Maternal vitamin D depletion alters DNA methylation at imprinted loci in multiple generations. Clin. Epigenetics.

[126] Junge K.M., Bauer T., Geissler S., Hirche F., Thurmann L., Bauer M., Trump S., Bieg M., Weichenhan D., Gu L. (2016). Increased vitamin D levels at birth and in early infancy increase offspring allergy risk-evidence for involvement of epigenetic mechanisms. J. Allergy Clin. Immunol..

[127] Suderman M., Stene L.C., Bohlin J., Page C.M., Holvik K., Parr C.L., Magnus M.C., Haberg S.E., Joubert B.R., Wu M.C. (2016). 25-Hydroxyvitamin D in pregnancy and genome wide cord blood DNA methylation in two pregnancy cohorts (MoBa and ALSPAC). J. Steroid Biochem. Mol. Biol..

[128] Benjamin Neelon S.E., White A.J., Vidal A.C., Schildkraut J.M., Murtha A.P., Murphy S.K., Kullman S.W., Hoyo C. (2018). Maternal vitamin D, DNA methylation at imprint regulatory regions and offspring weight at birth, 1 year and 3 years. Int. J. Obes..

[129] Reichetzeder C., Dwi Putra S.E., Li J., Hocher B. (2016). Developmental Origins of Disease-Crisis Precipitates Change. Cell. Physiol. Biochem..

[130] Zhou Y., Zhao L.J., Xu X., Ye A., Travers-Gustafson D., Zhou B., Wang H.W., Zhang W., Lee Hamm L., Deng H.W. (2014). DNA methylation levels of CYP2R1 and CYP24A1 predict vitamin D response variation. J. Steroid Biochem. Mol. Biol..

[131] Adams J.S., Hewison M. (2012). Extrarenal expression of the 25-hydroxyvitamin D-1-hydroxylase. Arch. Biochem. Biophys..

[132] Fransen F., van Beek A.A., Borghuis T., Meijer B., Hugenholtz F., van der Gaast-de Jongh C., Savelkoul H.F., de Jonge M.I., Faas M.M., Boekschoten M.V. (2017). The Impact of Gut Microbiota on Gender-Specific Differences in Immunity. Front. Immunol..

[133] Korpela K., Kallio S., Salonen A., Hero M., Kukkonen A.K., Miettinen P.J., Savilahti E., Kohva E., Kariola L., Suutela M. (2021). Gut microbiota develop towards an adult profile in a sex-specific manner during puberty. Sci. Rep..

[134] Wilmanski T., Diener C., Rappaport N., Patwardhan S., Wiedrick J., Lapidus J., Earls J.C., Zimmer A., Glusman G., Robinson M. (2021). Gut microbiome pattern reflects healthy ageing and predicts survival in humans. Nat. Metab..

[135] Yuan X., Chen R., Zhang Y., Lin X., Yang X. (2020). Gut microbiota: Effect of pubertal status. BMC Microbiol..

[136] Pelzer E., Gomez-Arango L.F., Barrett H.L., Nitert M.D. (2017). Review: Maternal health and the placental microbiome. Placenta.

[137] Buffington S.A., Di Prisco G.V., Auchtung T.A., Ajami N.J., Petrosino J.F., Costa-Mattioli M. (2016). Microbial Reconstitution Reverses Maternal Diet-Induced Social and Synaptic Deficits in Offspring. Cell.

[138] Kim S., Kim H., Yim Y.S., Ha S., Atarashi K., Tan T.G., Longman R.S., Honda K., Littman D.R., Choi G.B. (2017). Maternal gut bacteria promote neurodevelopmental abnormalities in mouse offspring. Nature.

[139] Vuong H.E., Pronovost G.N., Williams D.W., Coley E.J.L., Siegler E.L., Qiu A., Kazantsev M., Wilson C.J., Rendon T., Hsiao E.Y. (2020). The maternal microbiome modulates fetal neurodevelopment in mice. Nature.

[140] Dawson S.L., O’Hely M., Jacka F.N., Ponsonby A.L., Symeonides C., Loughman A., Collier F., Moreno-Betancur M., Sly P., Burgner D. (2021). Maternal prenatal gut microbiota composition predicts child behaviour. eBioMedicine.

[141] Aagaard K., Ma J., Antony K.M., Ganu R., Petrosino J., Versalovic J. (2014). The placenta harbors a unique microbiome. Sci. Transl. Med..

[142] Solt I. (2015). The human microbiome and the great obstetrical syndromes: A new frontier in maternal-fetal medicine. Best Pract. Res. Clin. Obstet. Gynaecol..

[143] DiGiulio D.B., Romero R., Amogan H.P., Kusanovic J.P., Bik E.M., Gotsch F., Kim C.J., Erez O., Edwin S., Relman D.A. (2008). Microbial prevalence, diversity and abundance in amniotic fluid during preterm labor: A molecular and culture-based investigation. PLoS ONE.

[144] DiGiulio D.B., Romero R., Kusanovic J.P., Gomez R., Kim C.J., Seok K.S., Gotsch F., Mazaki-Tovi S., Vaisbuch E., Sanders K. (2010). Prevalence and diversity of microbes in the amniotic fluid, the fetal inflammatory response, and pregnancy outcome in women with preterm pre-labor rupture of membranes. Am. J. Reprod. Immunol..

[145] Onderdonk A.B., Hecht J.L., McElrath T.F., Delaney M.L., Allred E.N., Leviton A., Investigators E.S. (2008). Colonization of second-trimester placenta parenchyma. Am. J. Obstet. Gynecol..

[146] Collado M.C., Isolauri E., Laitinen K., Salminen S. (2008). Distinct composition of gut microbiota during pregnancy in overweight and normal-weight women. Am. J. Clin. Nutr..

[147] Chen J.J., Zeng B.H., Li W.W., Zhou C.J., Fan S.H., Cheng K., Zeng L., Zheng P., Fang L., Wei H. (2017). Effects of gut microbiota on the microRNA and mRNA expression in the hippocampus of mice. Behav. Brain Res..

[148] Bale T.L. (2015). Epigenetic and transgenerational reprogramming of brain development. Nat. Rev. Neurosci..

[149] Mallick R., Duttaroy A.K. (2023). Epigenetic modification impacting brain functions: Effects of physical activity, micronutrients, caffeine, toxins, and addictive substances. Neurochem. Int..

[150] Di Simone N., Santamaria Ortiz A., Specchia M., Tersigni C., Villa P., Gasbarrini A., Scambia G., D’Ippolito S. (2020). Recent Insights on the Maternal Microbiota: Impact on Pregnancy Outcomes. Front. Immunol..

[151] Bhatia P., Chhabra S. (2018). Physiological and anatomical changes of pregnancy: Implications for anaesthesia. Indian J. Anaesth..

[152] Koren O., Goodrich J.K., Cullender T.C., Spor A., Laitinen K., Backhed H.K., Gonzalez A., Werner J.J., Angenent L.T., Knight R. (2012). Host remodeling of the gut microbiome and metabolic changes during pregnancy. Cell.

[153] Rinninella E., Raoul P., Cintoni M., Franceschi F., Miggiano G.A.D., Gasbarrini A., Mele M.C. (2019). What is the Healthy Gut Microbiota Composition? A Changing Ecosystem across Age, Environment, Diet, and Diseases. Microorganisms.

[154] Shin N.R., Whon T.W., Bae J.W. (2015). Proteobacteria: Microbial signature of dysbiosis in gut microbiota. Trends Biotechnol..

[155] Prochazkova N., Falony G., Dragsted L.O., Licht T.R., Raes J., Roager H.M. (2023). Advancing human gut microbiota research by considering gut transit time. Gut.

[156] Zakaria Z.Z., Al-Rumaihi S., Al-Absi R.S., Farah H., Elamin M., Nader R., Bouabidi S., Suleiman S.E., Nasr S., Al-Asmakh M. (2022). Physiological Changes and Interactions Between Microbiome and the Host During Pregnancy. Front. Cell. Infect. Microbiol..

[157] Rubini E., Schenkelaars N., Rousian M., Sinclair K.D., Wekema L., Faas M.M., Steegers-Theunissen R.P.M., Schoenmakers S. (2022). Maternal obesity during pregnancy leads to derangements in one-carbon metabolism and the gut microbiota: Implications for fetal development and offspring wellbeing. Am. J. Obstet. Gynecol..

[158] Manrique-Corredor E.J., Orozco-Beltran D., Lopez-Pineda A., Quesada J.A., Gil-Guillen V.F., Carratala-Munuera C. (2019). Maternal periodontitis and preterm birth: Systematic review and meta-analysis. Community Dent. Oral Epidemiol..

[159] Zakis D.R., Paulissen E., Kornete L., Kaan A.M.M., Nicu E.A., Zaura E. (2022). The evidence for placental microbiome and its composition in healthy pregnancies: A systematic review. J. Reprod. Immunol..

[160] Mousa W.K., Chehadeh F., Husband S. (2022). Microbial dysbiosis in the gut drives systemic autoimmune diseases. Front. Immunol..

[161] Wang J., Gu X., Yang J., Wei Y., Zhao Y. (2019). Gut Microbiota Dysbiosis and Increased Plasma LPS and TMAO Levels in Patients With Preeclampsia. Front. Cell. Infect. Microbiol..

[162] Nyangahu D.D., Jaspan H.B. (2019). Influence of maternal microbiota during pregnancy on infant immunity. Clin. Exp. Immunol..

[163] Hu Y., Jin P., Peng J., Zhang X., Wong F.S., Wen L. (2016). Different immunological responses to early-life antibiotic exposure affecting autoimmune diabetes development in NOD mice. J. Autoimmun..

[164] Holladay S.D., Smialowicz R.J. (2000). Development of the murine and human immune system: Differential effects of immunotoxicants depend on time of exposure. Environ. Health Perspect..

[165] Eberl G., Lochner M. (2009). The development of intestinal lymphoid tissues at the interface of self and microbiota. Mucosal Immunol..

[166] Westrom B., Arevalo Sureda E., Pierzynowska K., Pierzynowski S.G., Perez-Cano F.J. (2020). The Immature Gut Barrier and Its Importance in Establishing Immunity in Newborn Mammals. Front. Immunol..

[167] Holmes E., Li J.V., Marchesi J.R., Nicholson J.K. (2012). Gut microbiota composition and activity in relation to host metabolic phenotype and disease risk. Cell Metab..

[168] McElroy S.J., Weitkamp J.H. (2011). Innate Immunity in the Small Intestine of the Preterm Infant. Neoreviews.

[169] Timm S., Schlunssen V., Olsen J., Ramlau-Hansen C.H. (2017). Prenatal antibiotics and atopic dermatitis among 18-month-old children in the Danish National Birth Cohort. Clin. Exp. Allergy.

[170] Ortqvist A.K., Lundholm C., Halfvarson J., Ludvigsson J.F., Almqvist C. (2019). Fetal and early life antibiotics exposure and very early onset inflammatory bowel disease: A population-based study. Gut.

[171] Joly A., Leulier F., De Vadder F. (2021). Microbial Modulation of the Development and Physiology of the Enteric Nervous System. Trends Microbiol..

[172] Yang L.L., Millischer V., Rodin S., MacFabe D.F., Villaescusa J.C., Lavebratt C. (2020). Enteric short-chain fatty acids promote proliferation of human neural progenitor cells. J. Neurochem..

[173] Pessa-Morikawa T., Husso A., Karkkainen O., Koistinen V., Hanhineva K., Iivanainen A., Niku M. (2022). Maternal microbiota-derived metabolic profile in fetal murine intestine, brain and placenta. BMC Microbiol..

[174] Garzoni L., Faure C., Frasch M.G. (2013). Fetal cholinergic anti-inflammatory pathway and necrotizing enterocolitis: The brain-gut connection begins in utero. Front. Integr. Neurosci..

[175] Collado M.C., Rautava S., Aakko J., Isolauri E., Salminen S. (2016). Human gut colonisation may be initiated in utero by distinct microbial communities in the placenta and amniotic fluid. Sci. Rep..

[176] He Q., Kwok L.Y., Xi X., Zhong Z., Ma T., Xu H., Meng H., Zhao F., Zhang H. (2020). The meconium microbiota shares more features with the amniotic fluid microbiota than the maternal fecal and vaginal microbiota. Gut Microbes.

[177] Chin A.M., Hill D.R., Aurora M., Spence J.R. (2017). Morphogenesis and maturation of the embryonic and postnatal intestine. Semin. Cell Dev. Biol..

[178] Toure A.M., Landry M., Souchkova O., Kembel S.W., Pilon N. (2019). Gut microbiota-mediated Gene-Environment interaction in the TashT mouse model of Hirschsprung disease. Sci. Rep..

[179] Schneider U., Bode F., Schmidt A., Nowack S., Rudolph A., Dolker E.M., Schlattmann P., Gotz T., Hoyer D. (2018). Developmental milestones of the autonomic nervous system revealed via longitudinal monitoring of fetal heart rate variability. PLoS ONE.

[180] Rackaityte E., Halkias J., Fukui E.M., Mendoza V.F., Hayzelden C., Crawford E.D., Fujimura K.E., Burt T.D., Lynch S.V. (2020). Viable bacterial colonization is highly limited in the human intestine in utero. Nat. Med..

[181] Martinez K.A., Romano-Keeler J., Zackular J.P., Moore D.J., Brucker R.M., Hooper C., Meng S., Brown N., Mallal S., Reese J. (2018). Bacterial DNA is present in the fetal intestine and overlaps with that in the placenta in mice. PLoS ONE.

[182] Ostrea E.M., Bielawski D.M., Posecion N.C. (2006). Meconium analysis to detect fetal exposure to neurotoxicants. Arch. Dis. Child..

[183] Jasarevic E., Howard C.D., Misic A.M., Beiting D.P., Bale T.L. (2017). Stress during pregnancy alters temporal and spatial dynamics of the maternal and offspring microbiome in a sex-specific manner. Sci. Rep..

[184] Kimura I., Miyamoto J., Ohue-Kitano R., Watanabe K., Yamada T., Onuki M., Aoki R., Isobe Y., Kashihara D., Inoue D. (2020). Maternal gut microbiota in pregnancy influences offspring metabolic phenotype in mice. Science.

[185] Segarra M., Aburto M.R., Acker-Palmer A. (2021). Blood-Brain Barrier Dynamics to Maintain Brain Homeostasis. Trends Neurosci..

[186] Goasdoue K., Miller S.M., Colditz P.B., Bjorkman S.T. (2017). Review: The blood-brain barrier; protecting the developing fetal brain. Placenta.

[187] Saunders N.R., Liddelow S.A., Dziegielewska K.M. (2012). Barrier mechanisms in the developing brain. Front. Pharmacol..

[188] Yu K., Rodriguez M., Paul Z., Gordon E., Gu T., Rice K., Triplett E.W., Keller-Wood M., Wood C.E. (2021). Transfer of oral bacteria to the fetus during late gestation. Sci. Rep..

[189] Rodriguez J.M., Murphy K., Stanton C., Ross R.P., Kober O.I., Juge N., Avershina E., Rudi K., Narbad A., Jenmalm M.C. (2015). The composition of the gut microbiota throughout life, with an emphasis on early life. Microb. Ecol. Health Dis..

[190] Guzzardi M.A., Ait Ali L., D’Aurizio R., Rizzo F., Saggese P., Sanguinetti E., Weisz A., Pellegrini M., Iozzo P. (2019). Fetal cardiac growth is associated with in utero gut colonization. Nutr. Metab. Cardiovasc. Dis..

[191] Belkaid Y., Hand T.W. (2014). Role of the microbiota in immunity and inflammation. Cell.

[192] Mikhailidis D.P., Jeremy J.Y., Barradas M.A., Dandona P., Hutton R.A. (1985). Increases in platelet and red cell counts, blood viscosity, and arterial pressure during mild surface cooling. Br. Med. J..

[193] Ratsika A., Codagnone M.C., O’Mahony S., Stanton C., Cryan J.F. (2021). Priming for Life: Early Life Nutrition and the Microbiota-Gut-Brain Axis. Nutrients.

[194] Clarke G., Grenham S., Scully P., Fitzgerald P., Moloney R.D., Shanahan F., Dinan T.G., Cryan J.F. (2013). The microbiome-gut-brain axis during early life regulates the hippocampal serotonergic system in a sex-dependent manner. Mol. Psychiatry.

[195] Jena A., Montoya C.A., Mullaney J.A., Dilger R.N., Young W., McNabb W.C., Roy N.C. (2020). Gut-Brain Axis in the Early Postnatal Years of Life: A Developmental Perspective. Front. Integr. Neurosci..

[196] Mueller N.T., Bakacs E., Combellick J., Grigoryan Z., Dominguez-Bello M.G. (2015). The infant microbiome development: Mom matters. Trends Mol. Med..

[197] Sajdel-Sulkowska E.M. (2023). The Impact of Maternal Gut Microbiota during Pregnancy on Fetal Gut-Brain Axis Development and Life-Long Health Outcomes. Microorganisms.

[198] Bonnin A., Goeden N., Chen K., Wilson M.L., King J., Shih J.C., Blakely R.D., Deneris E.S., Levitt P. (2011). A transient placental source of serotonin for the fetal forebrain. Nature.

[199] Bonnin A., Levitt P. (2011). Fetal, maternal, and placental sources of serotonin and new implications for developmental programming of the brain. Neuroscience.

[200] Dicks L.M.T. (2022). Gut Bacteria and Neurotransmitters. Microorganisms.

[201] Momose-Sato Y., Sato K. (2016). Development of synaptic networks in the mouse vagal pathway revealed by optical mapping with a voltage-sensitive dye. Eur. J. Neurosci..

[202] Sachis P.N., Armstrong D.L., Becker L.E., Bryan A.C. (1982). Myelination of the human vagus nerve from 24 weeks postconceptional age to adolescence. J. Neuropathol. Exp. Neurol..

[203] Carabotti M., Scirocco A., Maselli M.A., Severi C. (2015). The gut-brain axis: Interactions between enteric microbiota, central and enteric nervous systems. Ann. Gastroenterol..

[204] Chaudhry T.S., Senapati S.G., Gadam S., Mannam H., Voruganti H.V., Abbasi Z., Abhinav T., Challa A.B., Pallipamu N., Bheemisetty N. (2023). The Impact of Microbiota on the Gut-Brain Axis: Examining the Complex Interplay and Implications. J. Clin. Med..

[205] Dash S., Syed Y.A., Khan M.R. (2022). Understanding the Role of the Gut Microbiome in Brain Development and Its Association With Neurodevelopmental Psychiatric Disorders. Front. Cell Dev. Biol..

[206] Rusch J.A., Layden B.T., Dugas L.R. (2023). Signalling cognition: The gut microbiota and hypothalamic-pituitary-adrenal axis. Front. Endocrinol..

[207] Farzi A., Frohlich E.E., Holzer P. (2018). Gut Microbiota and the Neuroendocrine System. Neurotherapeutics.

[208] Kapoor A., Dunn E., Kostaki A., Andrews M.H., Matthews S.G. (2006). Fetal programming of hypothalamo-pituitary-adrenal function: Prenatal stress and glucocorticoids. J. Physiol..

[209] Condon J., Gosden C., Gardener D., Nickson P., Hewison M., Howie A.J., Stewart P.M. (1998). Expression of type 2 11beta-hydroxysteroid dehydrogenase and corticosteroid hormone receptors in early human fetal life. J. Clin. Endocrinol. Metab..

[210] Howland M.A., Sandman C.A., Glynn L.M. (2017). Developmental origins of the human hypothalamic-pituitary-adrenal axis. Expert Rev. Endocrinol. Metab..

[211] Sheng J.A., Bales N.J., Myers S.A., Bautista A.I., Roueinfar M., Hale T.M., Handa R.J. (2020). The Hypothalamic-Pituitary-Adrenal Axis: Development, Programming Actions of Hormones, and Maternal-Fetal Interactions. Front. Behav. Neurosci..

[212] Ng P.C. (2000). The fetal and neonatal hypothalamic-pituitary-adrenal axis. Arch. Dis. Child Fetal. Neonatal Ed..

[213] van Dijk S.J., Zhou J., Peters T.J., Buckley M., Sutcliffe B., Oytam Y., Gibson R.A., McPhee A., Yelland L.N., Makrides M. (2016). Effect of prenatal DHA supplementation on the infant epigenome: Results from a randomized controlled trial. Clin. Epigenetics.

[214] Robinson S.L., Mumford S.L., Guan W., Zeng X., Kim K., Radoc J.G., Trinh M.-H., Flannagan K., Schisterman E.F., Yeung E. (2020). Maternal fatty acid concentrations and newborn DNA methylation. Am. J. Clin. Nutr..

[215] Saffari A., Shrestha S., Issarapu P., Sajjadi S., Betts M., Sahariah S.A., Tomar A.S., James P., Dedaniya A., Yadav D.K. (2020). Effect of maternal preconceptional and pregnancy micronutrient interventions on children’s DNA methylation: Findings from the EMPHASIS study. Am. J. Clin. Nutr..

[216] Bianchi M., Alisi A., Fabrizi M., Vallone C., Ravà L., Giannico R., Vernocchi P., Signore F., Manco M. (2019). Maternal Intake of n-3 Polyunsaturated Fatty Acids During Pregnancy Is Associated with Differential Methylation Profiles in Cord Blood White Cells. Front. Genet..

[217] Koemel N.A., Senior A.M., Dissanayake H.U., Ross J., McMullan R.L., Kong Y., Phang M., Hyett J., Raubenheimer D., Gordon A. (2022). Maternal dietary fatty acid composition and newborn epigenetic aging—a geometric framework approach. Am. J. Clin. Nutr..

[218] Chen L., Wagner C.L., Dong Y., Wang X., Shary J.R., Huang Y., Hollis B.W., Zhu H. (2020). Effects of Maternal Vitamin D3 Supplementation on Offspring Epigenetic Clock of Gestational Age at Birth: A Post-hoc Analysis of a Randomized Controlled Trial. Epigenetics.

[219] Caffrey A., Irwin R.E., McNulty H., Strain J.J., Lees-Murdock D.J., McNulty B.A., Ward M., Walsh C.P., Pentieva K. (2018). Gene-specific DNA methylation in newborns in response to folic acid supplementation during the second and third trimesters of pregnancy: Epigenetic analysis from a randomized controlled trial. Am. J. Clin. Nutr..

[220] Küpers L.K., Fernández-Barrés S., Mancano G., Johnson L., Ott R., Vioque J., Colombo M., Landgraf K., Tobi E.W., Körner A. (2022). Maternal Dietary Glycemic Index and Glycemic Load in Pregnancy and Offspring Cord Blood DNA Methylation. Diabetes Care.

[221] Lecorguillé M., Navarro P., Chen L.-W., Murrin C., Viljoen K., Mehegan J., Shivappa N., Hébert J.R., Kelleher C.C., Suderman M. (2023). Maternal and Paternal Dietary Quality and Dietary Inflammation Associations with Offspring DNA Methylation and Epigenetic Biomarkers of Aging in the Lifeways Cross-Generation Study. J. Nutr..

[222] Gonzalez-Nahm S., Mendez M., Robinson W., Murphy S.K., Hoyo C., Hogan V., Rowley D. (2017). Low maternal adherence to a Mediterranean diet is associated with increase in methylation at the MEG3-IG differentially methylated region in female infants. Environ. Epigenetics.

[223] House J.S., Mendez M., Maguire R.L., Gonzalez-Nahm S., Huang Z., Daniels J., Murphy S.K., Fuemmeler B.F., Wright F.A., Hoyo C. (2018). Periconceptional Maternal Mediterranean Diet Is Associated with Favorable Offspring Behaviors and Altered CpG Methylation of Imprinted Genes. Front. Cell Dev. Biol..

[224] Chiu Y.-H., Fadadu R.P., Gaskins A.J., Rifas-Shiman S.L., Laue H.E., Moley K.H., Hivert M.-F., Baccarelli A., Oken E., Chavarro J.E. (2021). Dietary fat intake during early pregnancy is associated with cord blood DNA methylation at IGF2 and H19 genes in newborns. Environ. Mol. Mutagen..

[225] Geraghty A.A., Sexton-Oates A., O’Brien E.C., Saffery R., McAuliffe F.M. (2020). Epigenetic Patterns in Five-Year-Old Children Exposed to a Low Glycemic Index Dietary Intervention during Pregnancy: Results from the ROLO Kids Study. Nutrients.

[226] Geraghty A.A., Sexton-Oates A., O’Brien E.C., Alberdi G., Fransquet P., Saffery R., McAuliffe F.M. (2018). A Low Glycaemic Index Diet in Pregnancy Induces DNA Methylation Variation in Blood of Newborns: Results from the ROLO Randomised Controlled Trial. Nutrients.

[227] Sasaki T., Kawamura M., Okuno C., Lau K., Riel J., Lee M.-J., Miller C. (2024). Impact of Maternal Mediterranean-Type Diet Adherence on Microbiota Composition and Epigenetic Programming of Offspring. Nutrients.

